# Transition from Dendritic to Cell-like Crystalline Structures in Drying Droplets of Fetal Bovine Serum under the Influence of Temperature

**DOI:** 10.1021/acs.langmuir.2c00019

**Published:** 2022-03-31

**Authors:** Marina Efstratiou, John R. E. Christy, Daniel Bonn, Khellil Sefiane

**Affiliations:** † Division of Pharmacy and Optometry, Faculty of Biology, Medicine and Health, The University of Manchester, Stopford Building, Oxford Road, Manchester M13 9PL, U.K.; ‡ Institute of Multiscale Thermofluids, School of Engineering, The University of Edinburgh, King’s Buildings, James Clerk Maxwell Building, Peter Guthrie Tait Road, King’s Buildings, Edinburgh EH9 3FD, U.K.; § Institute of Physics, University of Amsterdam, Science Park 904, 1098XH Amsterdam, The Netherlands

## Abstract

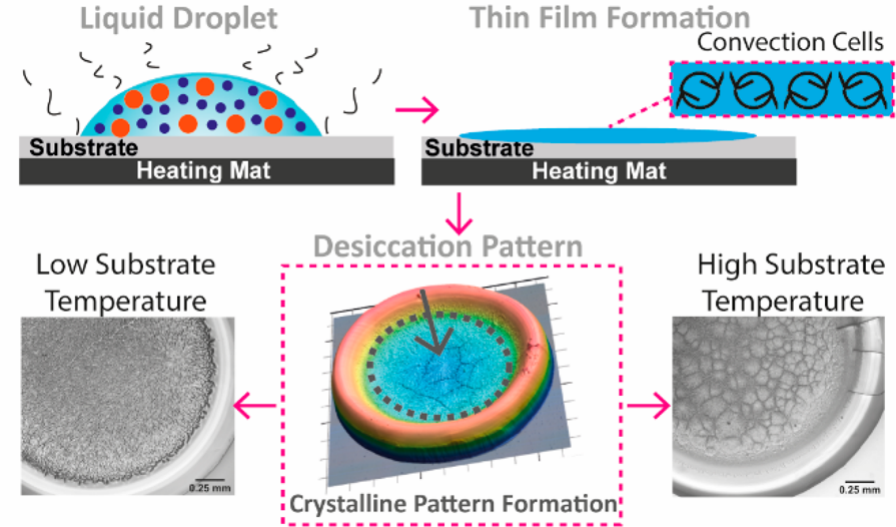

The desiccation of biofluid droplets leads to the formation of complex deposits which are morphologically affected by the environmental conditions, such as temperature. In this work, we examine the effect of substrate temperatures between 20 and 40 °C on the desiccation deposits of fetal bovine serum (FBS) droplets. The final dried deposits consist of different zones: a peripheral protein ring, a zone of protein structures, a protein gel, and a central crystalline zone. We focus on the crystalline zone showing that its morphological and topographical characteristics vary with substrate temperature. The area of the crystalline zone is found to shrink with increasing substrate temperature. Additionally, the morphology of the crystalline structures changes from dendritic at 20 °C to cell-like for substrate temperatures between 25 and 40 °C. Calculation of the thermal and solutal Bénard–Marangoni numbers shows that while thermal effects are negligible when drying takes place at 20 °C, for higher substrate temperatures (25–40 °C), both thermal and solutal convective effects manifest within the drying drops. Thermal effects dominate earlier in the evaporation process leading, we believe, to the development of instabilities and, in turn, to the formation of convective cells in the drying drops. Solutal effects, on the other hand, are dominant toward the end of drying, maintaining circulation within the cells and leading to crystallization of salts in the formed cells. The cell-like structures are considered to form because of the interplay between thermal and solutal convection during drying. Dendritic growth is associated with a thicker fluid layer in the crystalline zone compared to cell-like growth with thinner layers. For cell-like structures, we show that the number of cells increases and the area occupied by each cell decreases with temperature. The average distance between cells decreases linearly with substrate temperature.

## Introduction

Deegan’s paper in 1997, attributing the “coffee ring” effect to a capillary outward flow in droplets of suspensions or soluble solids,^1^ triggered wide interest in studying sessile drop evaporation of both pure^2,3^ and complex fluids^4−10^ as well as investigation of the patterns arising from drop dryout.^11−13^ For colloidal suspensions, because of continuity, the outward flow carries the particles to the contact line, causing the formation of a ring deposit. During the evaporation of colloidal drops, multiple hydrodynamic and physicochemical processes take place, which result in the formation of complex patterns.^14^ These processes include the spreading and adhesion of the drops to the substrate, contact line pinning, induced flows and component redistribution, crystallization, stress accumulation, and release.^15,16^ As a result of these processes, various morphologies have been reported within the final desiccation patterns, including rings, cracks, and crystalline regions.^11,17−20^

Biological fluids are considered to be complex colloidal systems, mainly composed of proteins, electrolytes, and water.^14,18^ During the desiccation of colloidal droplets, water evaporation causes the generation of surface tension gradients, which arise due to concentration gradients within the drying drops. In the case of a heated substrate, significant temperature gradients also arise because of the temperature difference between the warmer base of the drops, which is close to the substrate temperature, and the cooler apex of the drops, close to the ambient temperature. The development of concentration or temperature gradients causes convection and mixing within the droplet, leading to redistribution of the components. Because of the higher evaporation rate at the periphery of the drying drops, the solute concentration in that region increases, leading to supersaturation and preferential protein precipitation near the contact line, and hence, to the formation of an outer ring, mostly composed of proteins. Low molecular weight components, on the other hand, are observed to precipitate in the central region of the droplet. The evolution of the pattern over time and the final pattern formation, depend on the fluid composition.^21^

Understanding of the mechanisms involved during desiccation of biological fluid droplets, as well as of the emerging patterns, is of utmost importance for their application to medical diagnostics and forensic analysis.^22−25^ Studies have reported that changes in the composition of biological fluids, caused by disease, alter the final desiccation patterns.^26,27^ A multitude of studies have compared the final patterns arising from droplet evaporation of physiological and pathological biological fluids such as blood and blood serum.^23,24,28^ Additionally, Sobac and Brutin^29^ have described the different stages of blood droplet desiccation.

Because of the safety issues and difficulties involved in the acquisition of physiological fluids, many researchers have focused on the investigation of similar but simpler model systems, such as inorganic^19^ or organic^11,30^ colloids and colloidal solutions with salt admixtures. Various structures are observed when different types of ions are added to bovine serum albumin (BSA) solutions due to the interactions taking place within the solutions,^31^ leading to droplet spreading, gelation, crystallization, and crack formation.^17^ Pathak et al. have investigated how the addition of different salts (KCl and MgCl_2_) in aqueous BSA droplets affects the final desiccation patterns.^32^ Tarasevich and Pravoslavnova have developed a model for the evaporation of biological fluid drops, taking into account both diffusion and evaporative capillary flows.^33^

The desiccation of droplets consisting of biological fluids and the morphology of the final desiccation deposits depend on multiple factors including the type and concentration of macromolecules and electrolytes^31,34^ within the solution, the substrate properties such as wettability,^11,23,35^ and the ambient conditions under which evaporation takes place.^36,37^ Carreón et al.^38^ studied the effects of substrate temperature on the desiccation patterns of protein mixtures and protein–salt mixtures to find the most suitable temperature for efficient diagnosis.

The aforementioned studies offer a basis for the understanding of the impact of substrate temperature on the drying of protein–protein and protein–salt mixtures. Nevertheless, blood serum is a very complex mixture which consists of various types of macromolecules, electrolytes, antibodies, antigens, and hormones. Because of the complexity of biological fluids such as blood and serum, further research is required to gain a better understanding of the mechanisms involved in the desiccation of such systems. Additionally, the pH of the mixtures, which has a significant effect on protein conformation and aggregation by altering the electrostatic repulsive and attractive van der Waals forces, was not controlled in these studies. By affecting the interactions between the components, the pH can significantly influence the magnitude of the intermolecular forces and can therefore alter the final desiccation patterns of the dried drops. In this work, we use fetal bovine serum (FBS) drops to examine how substrate temperatures between 20 and 40 °C affect the final dried patterns. This temperature range was investigated as it is the most relevant in diagnostic applications. Compared to previous studies focusing on image analysis of the desiccation patterns of protein–protein and salt–protein mixtures, in this study we focus on the effect of substrate temperature on the morphology and topography of the central crystalline zone in the dried deposits and the phenomena affecting the pattern formation in this zone.

## Experimental Section

Fetal bovine serum (FBS South American A3160802, Thermofisher Scientific, UK) was received frozen in dry ice and used after defrosting and gentle mixing at room temperature. It should be noted that no vortex mixers or sonicators were used to avoid protein denaturation. FBS is a complex solution consisting of multiple proteins (Table 1), ions, and hormones. The ionic strength and pH of the solution are ∼0.14 M and 7.4, respectively.

**Table 1:** Type and Concentration of Proteins in the FBS Used in the Experiments

type of protein	typical concentration range	concentration in our sample
BSA	17–35 mg/mL	23 mg/mL
α-globulin	7–20 mg/mL	16 mg/mL
β-globulin	3–9 mg/mL	3.6 mg/mL
γ-globulin	10–200 μg/mL	23.73 μg/mL
hemoglobin	0.01–0.30 mg/mL	0.1401 mg/mL

Glass microscope slides (MS/1 Scientific Glass Laboratories Ltd., UK) were placed in an ultrasonic bath with deionized water for 15 min and then rinsed with ethanol (ethanol, 99%+, Absolute, Fisher Scientific, UK) and dried by using an air gun. Sessile drops of FBS (1.2 ± 0.2 μL) were gently placed on the glass slides and left to dry at different substrate temperatures. The initial contact angle between the liquid FBS droplets and the substrates was 35 ± 5°. The substrate temperature was controlled with the use of a heater mat (SRFRA-4/10-230V, Omega Engineering Ltd., UK), connected to a PID controller that enabled the temperature adjustments, placed underneath the glass surfaces, and a thermocouple mounted on the slide surface (Figure S1 in the Supporting Information). Both the ambient temperature and the relative humidity (RH) levels were monitored during the experimental procedure via the use of a temperature–humidity meter (HH311, Omega Engineering Ltd., UK). RH levels were 45 ± 5% during the experiments, and the ambient temperature was 20 ± 1 °C.

The temporal evolution of droplet volume, diameter, and contact angle were monitored with the use of a goniometer (DSA-30S Drop Shape Analyzer, KRÜSS, Germany) which enabled the investigation of the side-profile evolution of the drops. Images were captured after completion of the desiccation process under different magnifications (2×, 5×, 10×) for the investigation of the final deposition patterns. Top view images of the droplets were also captured during desiccation using an optical microscope (Euromex, Netherlands) connected to a CMOS camera (MAKO G-507 Allied Vision Technologies, UK) under 2× magnification. All the deposits were also examined 24 h after the completion of the experiments.

3D topographical studies were also conducted on the desiccated deposits. A confocal laser scanning microscope (VK-X1000, Keyence, Netherlands) was utilized to obtain 3D topographs, in combination with the Keyence Multifile Analyzer software for height measurements in different zones of the dried deposits. Topographical data were acquired under 20× magnification. Because the view of the entire desiccation pattern was challenging under such high magnification, multiple regions of each drop were examined separately, and then stitched together in Keyence Multifile Analyzer, to provide the topography of the final desiccation pattern. The experimental procedure was similar as described above by using the goniometer. Topographical investigation of the deposits allowed measurements of the average thickness (height) and radius of each region of the desiccation patterns. Based on the measured radius, the area of each deposit was estimated. This allowed the calculation of the final deposit volume and the volume of each zone within the dried deposits, based on measurements of thickness and radius.

Each set of experiments was repeated at least three times for each substrate temperature. The final deposits showed good reproducibility regarding the final morphology for each of the temperatures in all of these methods.

## Results and Discussion

**Figure 1:**
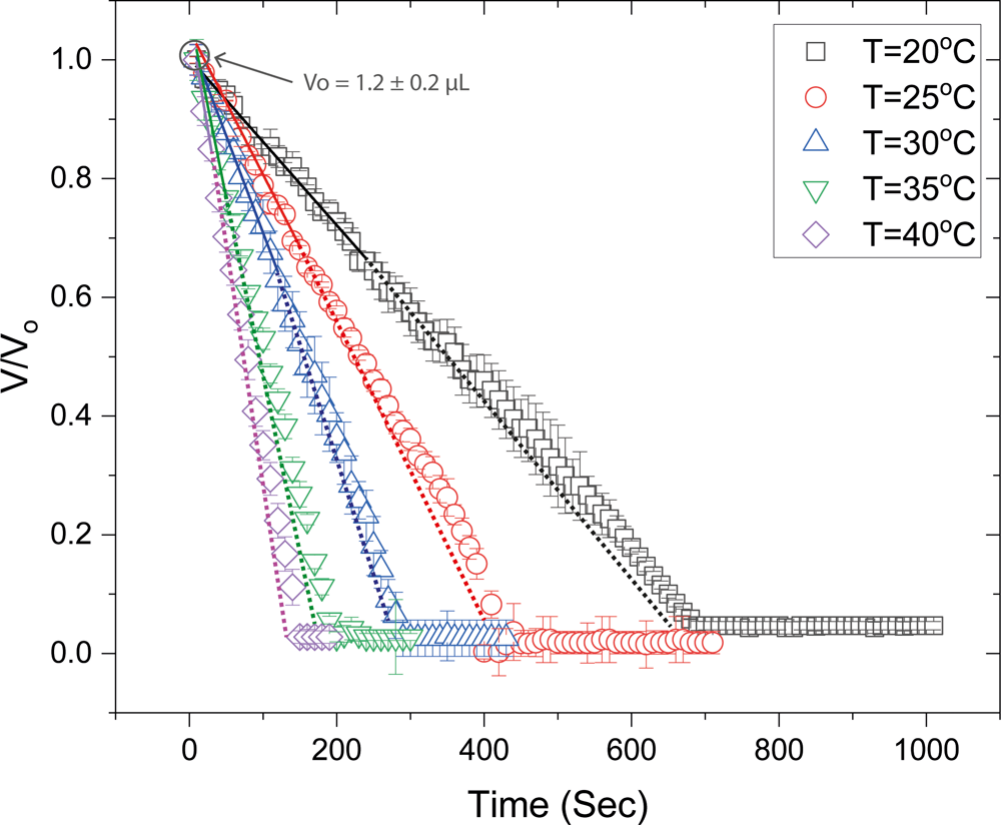
Evolution of the droplet volume for FBS droplets drying on glass slides of different temperature. *V*_0_ is the initial drop volume at the onset of drying. The drop volume decreases in a linear manner until the onset of gelation (continuous lines). Dashed lines show the extrapolation of the evaporation kinetics according to the evaporation rate at the early stage of pregelation. The average times for the onset of gelation are ∼250, ∼163, ∼129, ∼66, and ∼38 s for droplets evaporating on glass slides with temperature 20, 25, 30, 35, and 40 °C, respectively. It should be noted that the volume data points appear to asymptote to a constant value during crystallization and cracking. This is due to limitations of the experimental apparatus to make accurate measurements when the remaining volume is very small. The final volume of the deposits is in the order of tens of nanoliters.

Four desiccation stages were identified during drying for all the examined substrate temperatures. These are pregelation, gelation, crystallization and crack formation. During pregelation, the drying is dominated by water evaporation at the contact line where the evaporation rate is higher. Because of the higher evaporation rate at the periphery, a radially outward capillary flow develops, carrying fluid toward the contact line. This leads to the aggregation and precipitation of proteins at the contact line and induces pinning of the drop to the substrate. Protein adsorption follows a complex series of adsorption–displacement steps in which proteins of lower molecular weight adsorb to the substrate first and are then displaced by proteins of higher molecular weights. This phenomenon is called the “Vroman effect”.^39^ Because of pinning, evaporation proceeds in a constant contact radius (CCR) mode, during which the contact angle decreases over time. The droplet volume decreases linearly over time, indicating a constant evaporation rate, until the onset of gelation (continuous lines), after which a small decrease of the evaporation rate is observed, as shown in Figure 1. The dashed lines in Figure 1 show the extrapolation of the initial evaporation kinetics of the droplets, based on the evaporation rate at the early stage of pregelation. The precipitation of proteins at the contact line leads to gelation, during which a sol–gel transition occurs. Gelation propagates from the periphery toward the center of the drying drops. The width of the gel region increases with time. The gel is a porous film that traps water, causing the evaporation rate to decrease, as water molecules have to diffuse through the film to escape to vapor.^40^ After the completion of gelation, crystallization and cracking take place. Crystallization takes place in the central region of the formed gel, giving rise to the formation of crystalline structures. Almost simultaneously (exactly upon the onset of crystal nucleation or within a few seconds), cracking occurs on the peripheral protein ring. Cracking lasts longer than the crystallization process. No volume changes are observed throughout the duration of crystallization and cracking due to limitations of the experimental apparatus to make accurate measurements when the remaining volume is very small (Figure 1). It is noteworthy that the final volume of the deposits is in the order of tens of nanoliters.

### Effect of Temperature on the Morphology of the Final Deposits

**Figure 2:**
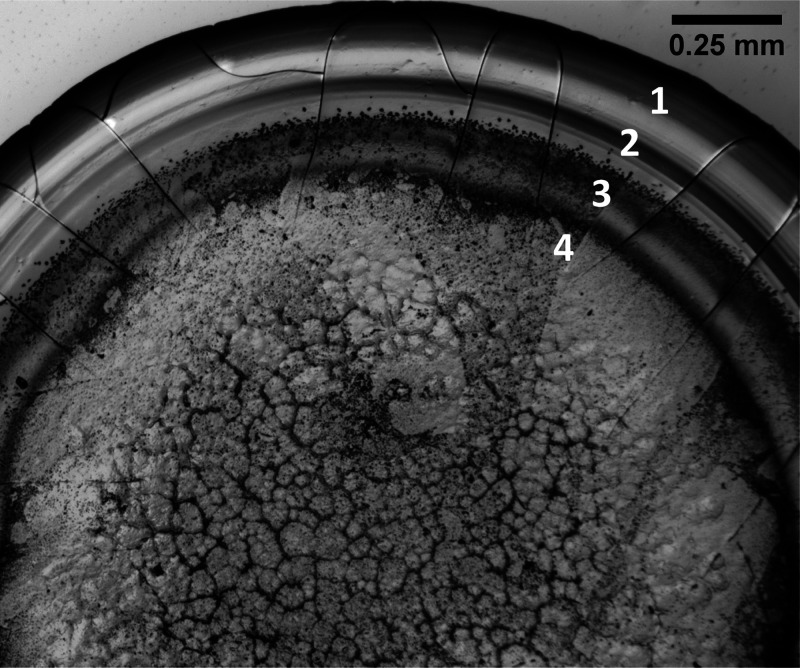
Four distinct zones are observed in the desiccation patterns of FBS droplets: (1) the peripheral ring where cracking occurs, (2) clusters of protein structures, (3) the protein gel, and (4) the central crystalline zone. The image was acquired under 5× magnification after desiccation of a FBS drop on a glass slide heated at 40 °C, 24 h after the completion of the experiment.

It was shown in a previous work^26^ that for aqueous drops of 7% BSA–0.9% NaCl solution, four distinct zones are observed in the desiccation deposits after evaporation. Moving from the edge of the drop toward the center, these zones are (1) a zone of homogeneous protein (peripheral ring), (2) a zone of protein structures, (3) the protein gel, and (4) the crystalline zone within the protein gel. The proteins exist at different conditions within each zone, forming materials of different properties: a glassy (high volume fraction) protein ring on the periphery and a protein gel (lower volume fraction) in the interior of the ring. In the central area of the desiccated drops, the volume fraction of proteins is lower, but the ionic strength increases.^26^ The same zones were observed in our experiments, with the zones of protein structures and gel being more evident between 25 and 40 °C. The different zones for the desiccated deposits are shown in Figure 2.

### Gel and Central Crystalline Zone

**Figure 3:**
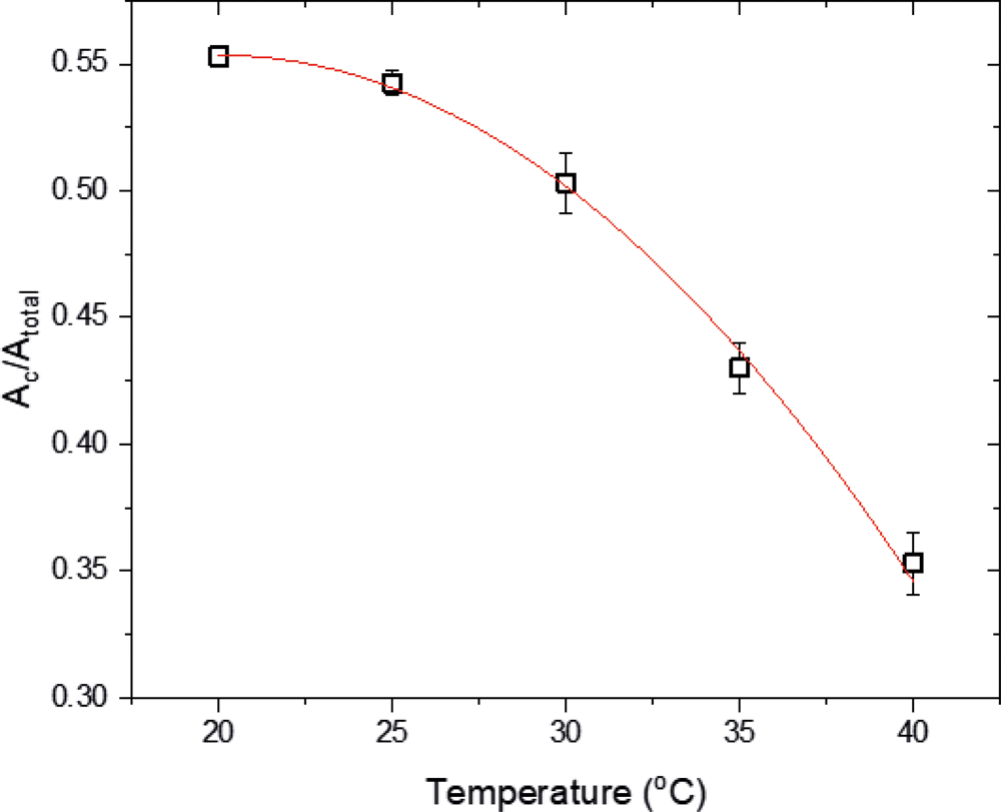
Graph showing the ratio of the crystalline area to the entire deposit area (*A*_c_/*A*_total_) versus temperature. The best fit line (red) is approximated by a second-order polynomial curve.

**Figure 4:**
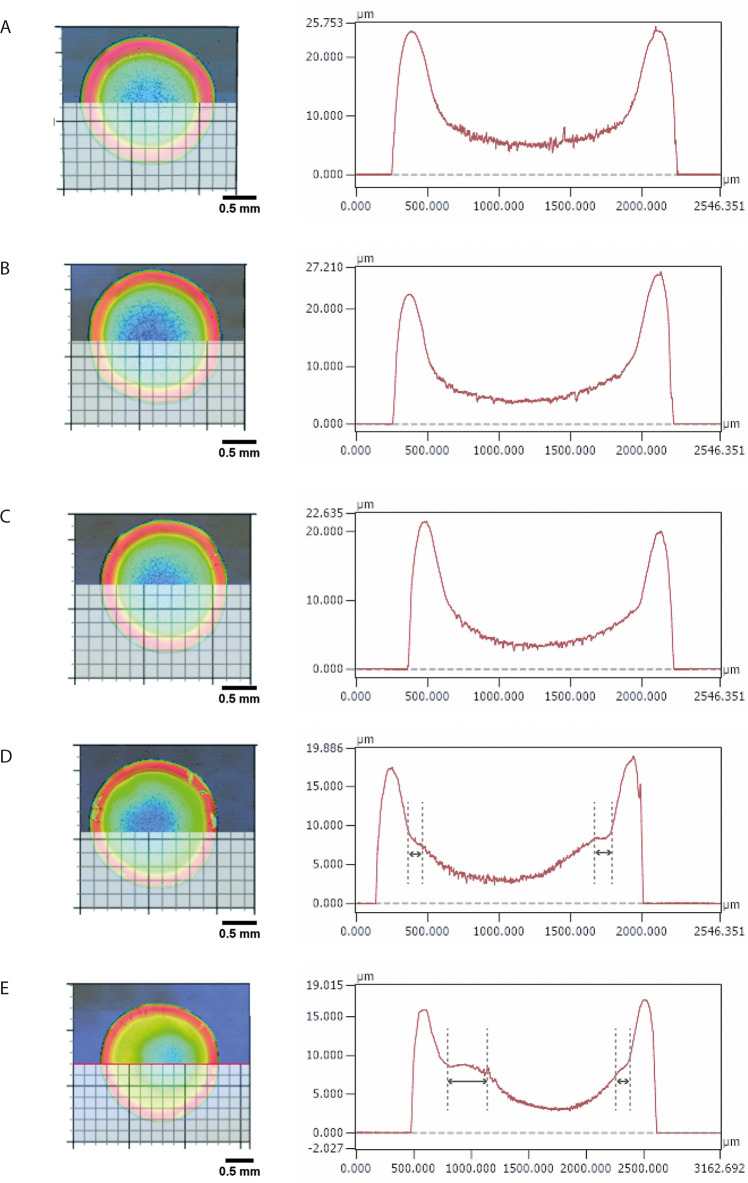
Left: top view images of the deposits illustrating the horizontal line along which the height profile is shown. Right: height profile of the deposits occurring after droplet drying at (A) 20, (B) 25, (C) 30, (D) 35, and (E) 40 °C. The gray arrows indicate the increase in the width of the gel region for droplets evaporating at higher temperatures (35 and 40 °C).

Increasing the substrate temperature causes the central crystalline zone in the desiccation patterns to shrink. This is shown both on the microscopic images acquired after evaporation and on the 3D topographical data acquired via confocal laser scanning microscopy (Figure S2). Image analysis showed that variation of the ratio of the crystalline area (*A*_c_) to the total area of the desiccation deposit (*A*_total_) with temperature can be approximated by a second-order polynomial curve (Figure 3). The reason for this is not clear yet. Because of the shrinking of the crystalline zone, the width of the gel between the glassy protein ring and the crystalline zone increases with temperature. The increase in the width of the protein gel is accompanied by a decreasing height with increasing temperature. The gel is more profound at the highest examined temperatures of 35 and 40 °C (Figure 4 and Figure S3). The glassy peripheral protein ring appears to be the thickest feature of the desiccation patterns for the range of temperatures examined in this work. Topographical investigation has shown that the height of the deposit decreases from the peripheral protein ring (red) toward the region of protein aggregates and the gel (yellow and green) and the central crystalline zone which appears to be the thinnest feature as shown in Figure 4.

**Figure 5:**
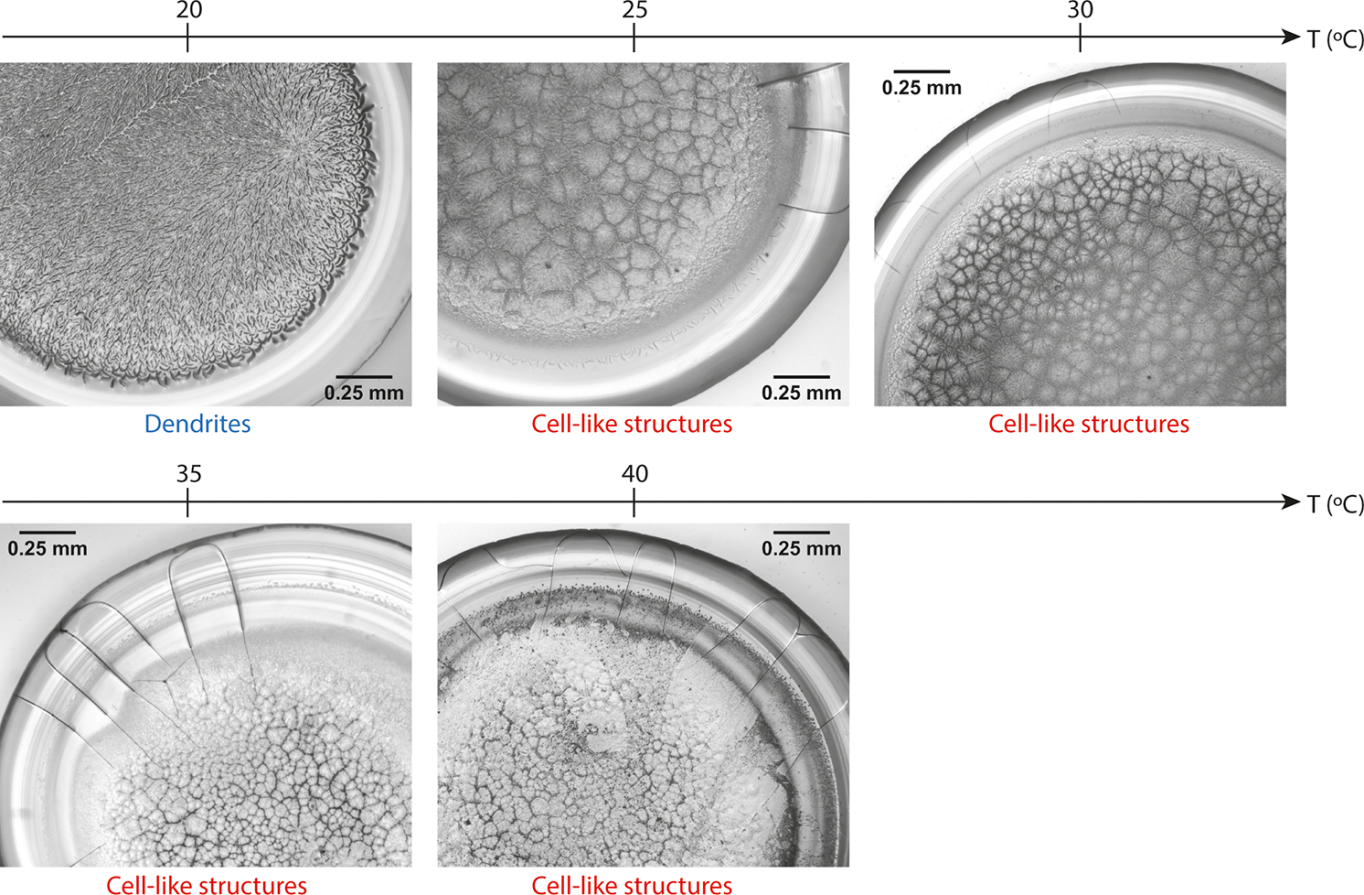
Final desiccation deposits illustrating different crystalline morphological features, depending on the substrate temperature. For droplets drying at 20 °C, the central crystalline zone consists of dendrites, whereas for higher substrate temperatures, it is composed of cell-like structures, which become finer as the temperature increases.

The temperature increase appears to have an effect on the morphology of the final crystalline structures forming in the central region of the dried deposit, as shown in Figure 5. The morphology of the crystals is probably related to the thickness of the crystalline zone, as will be discussed further later. Although for droplets evaporating at 20 °C the final crystalline deposit consists of dendritic structures, at higher temperatures, the central region is composed of cell-like structures, which become finer with increasing temperature from 25 to 40 °C. The time for the onset of crystal nucleation and the duration of crystallization differ for each of the examined temperatures. Dendrite nucleation is initiated at approximately 60% of the droplet lifetime, whereas cell-like structures start to form at ∼78% of the lifetime for droplets drying at 25 °C (Figure S4). For cell-like structures, the time at which crystallization commences, as well as its duration, decreases with increasing temperature because the evaporation rate varies with temperature.

**Figure 6:**
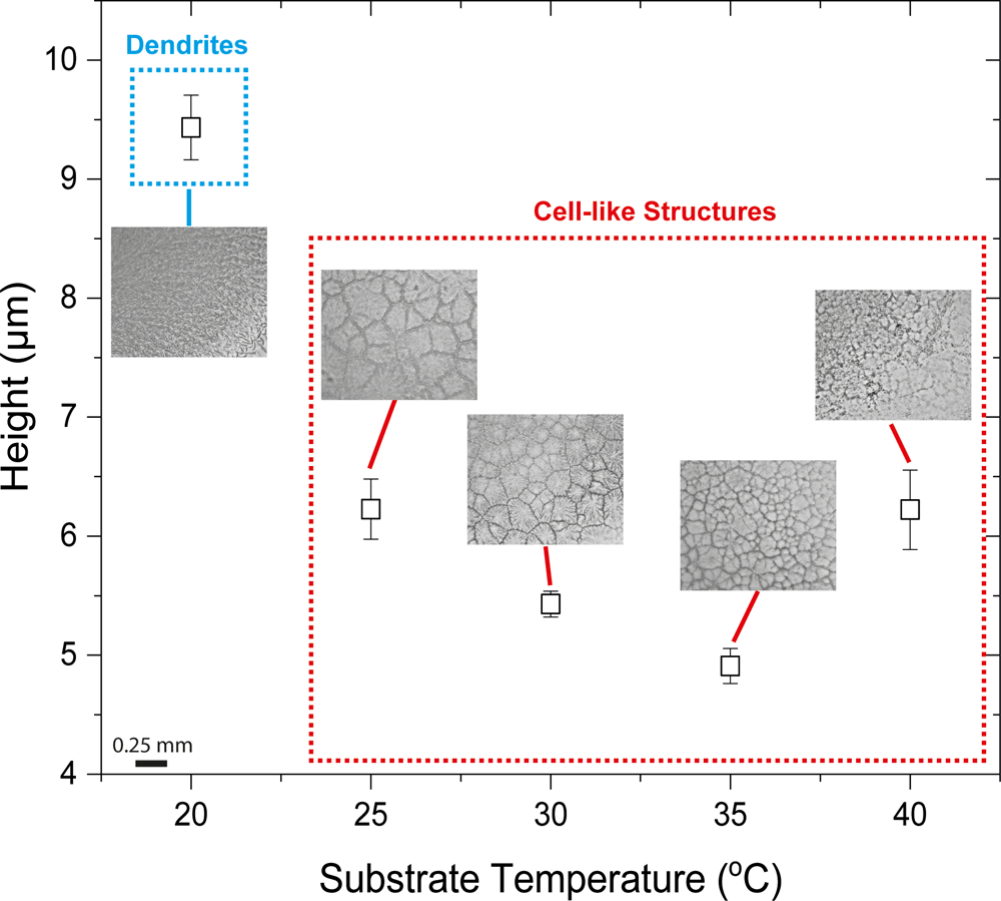
Final height of the central crystalline zone in the desiccation patterns with substrate temperature. Insets show the morphology of the crystalline zone for each substrate temperature. In the case of dendrites, a thicker film forms, whereas for cell-like structures a thinner film is observed.

In an attempt to probe the effect of temperature on the formation of crystalline structures, we proceed to the investigation of the central crystalline zone. Investigation of the desiccation deposits via confocal laser scanning microscopy enables the determination of the average height (thickness) and area of each zone within the deposits. Focusing on the central crystalline zone, we measure the final average thickness of this region with substrate temperature (Figure 6). The thickness of the central crystalline zone varies significantly in the case of dendrites, formed on substrates at 20 °C, compared to the case of cell-like structures, formed on substrates of higher temperatures (25–40 °C). The average final thickness in the case of dendritic formation is approximately 9.5 μm, whereas for cell-like structures it varies between ∼4.9 and ∼6.2 μm, depending on the substrate temperature. The final thickness decreases from approximately 6.2 μm at 25 °C to ∼5.4 and ∼4.9 μm at 30 and 35 °C, respectively, and it increases from 35 to 40 °C (∼6.2 μm at 40 °C). This indicates that the thickness of the central crystalline zone is significantly thicker when drying takes place on a substrate with temperature of 20 °C compared to higher temperatures, i.e., 25–40 °C. This finding suggests that the lower evaporation rate at lower substrate temperatures leads to the formation of a thicker film in the central region of the dried deposit, manifesting dendritic structures. The thinner film forming in the central region at higher temperatures, on the other hand, consists of cell-like crystalline structures. Therefore, the type of crystalline structures occurring could be related to thickness of the central region.

### Hydrodynamic and Intermolecular Forces Acting within the Droplets at the Onset of Evaporation

In an attempt to understand the phenomena governing the pattern formation, we have performed an approximate analysis of the hydrodynamic (capillary, drag) and intermolecular forces acting in the droplets at the onset of drying (see the Supporting Information). To do this, we considered the spherical-like geometry of the protein macromolecules in fetal bovine serum.^41−43^ The approach for the calculation of hydrodynamic and intermolecular forces is similar to that employed in other studies (32, 44, and 45), treating proteins as spherical macromolecules. The equations used for the estimation of electrostatic and van der Waals forces on macromolecules may also be found in other works (46 and 47). This analysis suggests that the hydrodynamic fluid forces dominate initially, while macromolecules are in solution, but that once deposition starts, at the molecular level intermolecular attractive protein–protein and protein–substrate forces dominate over electrostatic forces, which may partially explain why the salts tend to diffuse out from the protein deposited at the contact line. Capillary forces acting on protein macromolecules (*O*(10^–9^ N)) are found to be orders of magnitude higher compared to drag forces or intermolecular forces. The attractive protein interactions between proteins of the same type are in the order of *O*(10^–14^ N) at 25 °C. The order of magnitude of these forces varies from *O*(10^–14^ N) to *O*(10^–15^ N), depending on the type of biomolecule, when the substrate temperature is 40 °C. Protein–substrate interactions may increase from *O*(10^–15^ N) to *O*(10^–14^ N) or drop from *O*(10^–14^ N) to *O*(10^–15^ N) for different types of proteins, when the substrate temperature changes from 25 to 40 °C.

If we analyze the hydrodynamic fluid forces in the central region following deposition of proteins at the outer edge, these will be influenced by the temperature difference between the heated substrate and the liquid vapor surface at which evaporation takes place. We suspect that the pattern formation in the central crystalline region might be related to a thin film phenomenon. After gelation, the central region of the drying drop does not have a hemispherical shape anymore, but it resembles a thin film instead due to water evaporation. When the substrate is close to room temperature, no significant temperature gradients are expected within the film, and we do not anticipate any significant thermally driven surface temperature gradients. With a lack of driving force we expect the salt concentration in the central region to increase until the level of supersaturation results in nucleation of a crystal, with subsequent crystallization extending from the one nucleation site.

In the case of the heated substrate, we expect significant temperature gradients to develop initially between the base of the drop and the apex of the drop. The formation of cell-like structures for temperatures between 25 and 40 °C may be attributed to thermal Bénard–Marangoni convection, leading to the development of instabilities and resulting in the formation of polygonal (hexagonal-like) convective cells within the drying liquid film.^48^ To probe whether the cell-like formation could be due to the development of instabilities, we proceed to investigate the convective effects for the substrate temperatures examined in this study.

### Bénard–Marangoni Convection

For small droplets deposited on substrates with *h* ≪ *R*, the gravitational effects may be neglected because of the small Bond number . In this case, convection is driven by surface tension effects (Bénard–Marangoni) rather than buoyancy effects (Rayleigh–Bénard). Bénard–Marangoni convection develops due to a surface tension gradient on the free surface.^48^

This surface tension gradient may result from a temperature gradient (thermal Bénard–Marangoni) and/or a concentration gradient (solutal Bénard–Marangoni). The effect of thermal and solutal Bénard–Marangoni instabilities may be evaluated by calculation of the dimensionless thermal (*Ma*_*T*_) and solutal (*Ma*_*S*_) Bénard–Marangoni numbers, respectively.

The thermal Bénard–Marangoni number is defined as
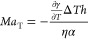
1where  is the change of the surface tension with temperature, Δ*T* is the temperature difference at the air/liquid interface, *h* is the film thickness (height), η is the dynamic viscosity of the fluid, and α is the thermal diffusivity. Thermal Bénard–Marangoni instabilities develop because of a temperature gradient on the free surface, giving rise to a surface tension gradient.

The solutal Bénard–Marangoni number, on the other hand, is given by
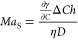
2where  is the change of the surface tension with concentration, Δ*C* is the concentration difference at the air/liquid interface, and *D* is the diffusion coefficient. Solutal Bénard–Marangoni instabilities may develop from a concentration gradient on the free surface. The solvent evaporation from the free surface can lead to the development of a concentration gradient across the air/liquid interface, between the base and the apex.

The surface tension gradients arising at the air/liquid interface from thermal or solutal effects give rise to the development of internal flows in the liquid film. The strength of these flows and the velocities developing in the fluid are related to the magnitude of Bénard–Marangoni numbers; hence, we estimate the maximum values that these numbers can take in our system for the temperatures studied. For the estimation of the thermal (*Ma*_*T*_) Bénard–Marangoni numbers we assume the temperature difference at the free surface with the base having the temperature of the heated substrate, whereas the apex is at the ambient temperature (20 °C). The critical thermal Marangoni number *Ma*_*T*_^*C*^ for the formation of convective thermal Bénard–Marangoni instabilities has been estimated to be ∼80 for water films.^49,50^

The values of parameters for the calculations of *Ma*_*S*_ and *Ma*_*T*_ are given in the Supporting Information (Table S5). Because of the preferential deposition of proteins at the contact line, the volume fraction of proteins is higher at the droplet edge, whereas the volume fraction of proteins is lower in the central region.^26,51−53^ As a result, the viscosity increases locally near the peripheral ring due to protein deposition. However, the central region of the droplets is liquid resembling a thin film in which the volume fraction of proteins is relatively low and the viscosity of the fluid film remains close to the initial viscosity of FBS. For the calculations of Bénard–Marangoni numbers we use the height of the drying droplets at the end of gelation when the thin film has completely formed and crystallization has not occurred yet. Data on the height of the drying drops have been obtained during the evaporative process. Figure S5 shows the evolution of the normalized height during drying. The height has been normalized based on the initial height values at the onset of drying (∼0.45 mm).

**Figure 7:**
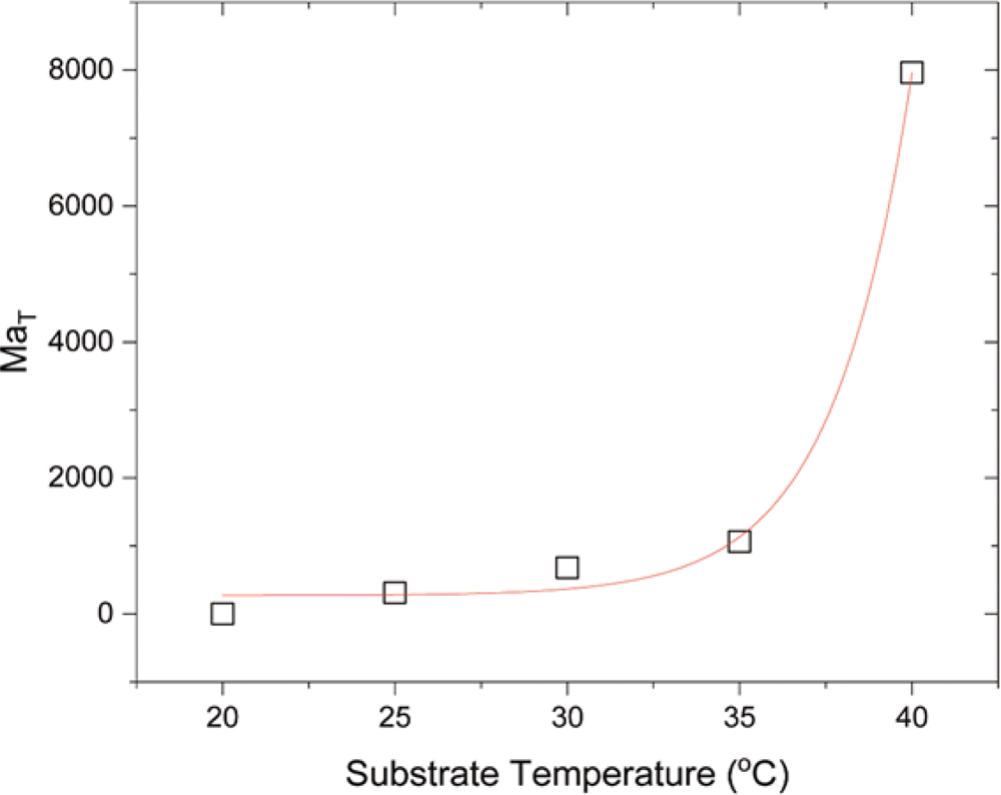
Estimation of thermal (*Ma*_*T*_) Bénard–Marangoni numbers at the end of gelation for different substrate temperatures.

During the early evaporation stages (prior to gelation), the salt concentration difference between the apex and the base of the drying drops is small, which results in low *Ma*_*S*_. On the other hand, *Ma*_*T*_ is higher than *Ma*_*S*_ during the early evaporation stages. This suggests that thermal Bénard–Marangoni convection dominates earlier in the evaporation process when the convective cells set up within the drying film. The temporal evolution of *Ma*_*T*_ has been estimated for droplets drying on substrates of different temperatures (Figure S6). Figure 7 shows the calculated thermal Bénard–Marangoni numbers at the end of gelation for each examined substrate temperature. When the substrate temperature is 20 °C, no significant temperature gradients arise, and the thermal Bénard–Marangoni number is approximately zero. For higher substrate temperatures, however, thermal Bénard–Marangoni numbers increase significantly to ∼308, ∼677, ∼1064, and ∼7965 for substrate temperatures of 25, 30, 35, and 40 °C, respectively. The estimation of such high thermal Bénard–Marangoni numbers at the end of gelation for higher substrate temperatures supports our hypothesis that the cell-like structures are related to a strong thermal Bénard–Marangoni convection in the drying drops, giving rise to instabilities and the formation of convective cells. A high temperature gradient exists across the film causing the development of instabilities and leading to the formation of convective cells. Within these cells the fluid rises at the air/liquid interface, cools and moves downward at the center and out along the base. Thus, the temperature gradient leads to thermal Bénard–Marangoni convection, creating toroidal vortices within each cell.

Although thermal Bénard–Marangoni convection appears to be responsible for the setup of the convective cells early in the evaporation process defining the number and the shape of the cells, it is possible that as drying proceeds, the maintenance of circulation in the cells is enhanced by solutal Bénard–Marangoni convection. At the latter stages of evaporation, convective cells are fully formed in the drying film. We believe that the toroidal vortices formed within each cell by thermal Bénard–Marangoni effects result in more concentrated solution flowing upward to the air/liquid interface of each cell. As drying proceeds, the fluid is carried upward at the edge of the cell to the air/liquid interface, reaching supersaturation at the surface and leading to crystal nucleation in the center of the base of each cell. Once nucleation commences, the fluid moving upward the edge of the cell is saturated rather than supersaturated. This internal circulation, initiated by thermal effects, is sustained by solutal effects, since surface tensions are higher for both lower temperatures and higher salt concentrations, maintaining the toroidal flow patterns within each cell. For the calculations of the solutal (*Ma*_*S*_) Bénard–Marangoni numbers, we consider that Δ*C* = *C*_ssat_ – *C*_sat_, where *C*_ssat_ is the supersaturation concentration (at the apex) and *C*_sat_ is the saturation concentration (at the base). The estimated solutal Bénard–Marangoni numbers at the end of gelation just prior to the onset of crystallization are approximately 6.2 × 10^4^, 6.8 × 10^4^, 7.1 × 10^4^, and 39.9 × 10^4^ for 25, 30, 35, and 40 °C, respectively. In all cases, we believe the existence of the cells to be related to surface tension effects, with thermal Bénard–Marangoni convection being responsible for the cell formation and solutal Bénard–Marangoni convection being responsible for the maintenance of cells during crystallization.

**Figure 8:**
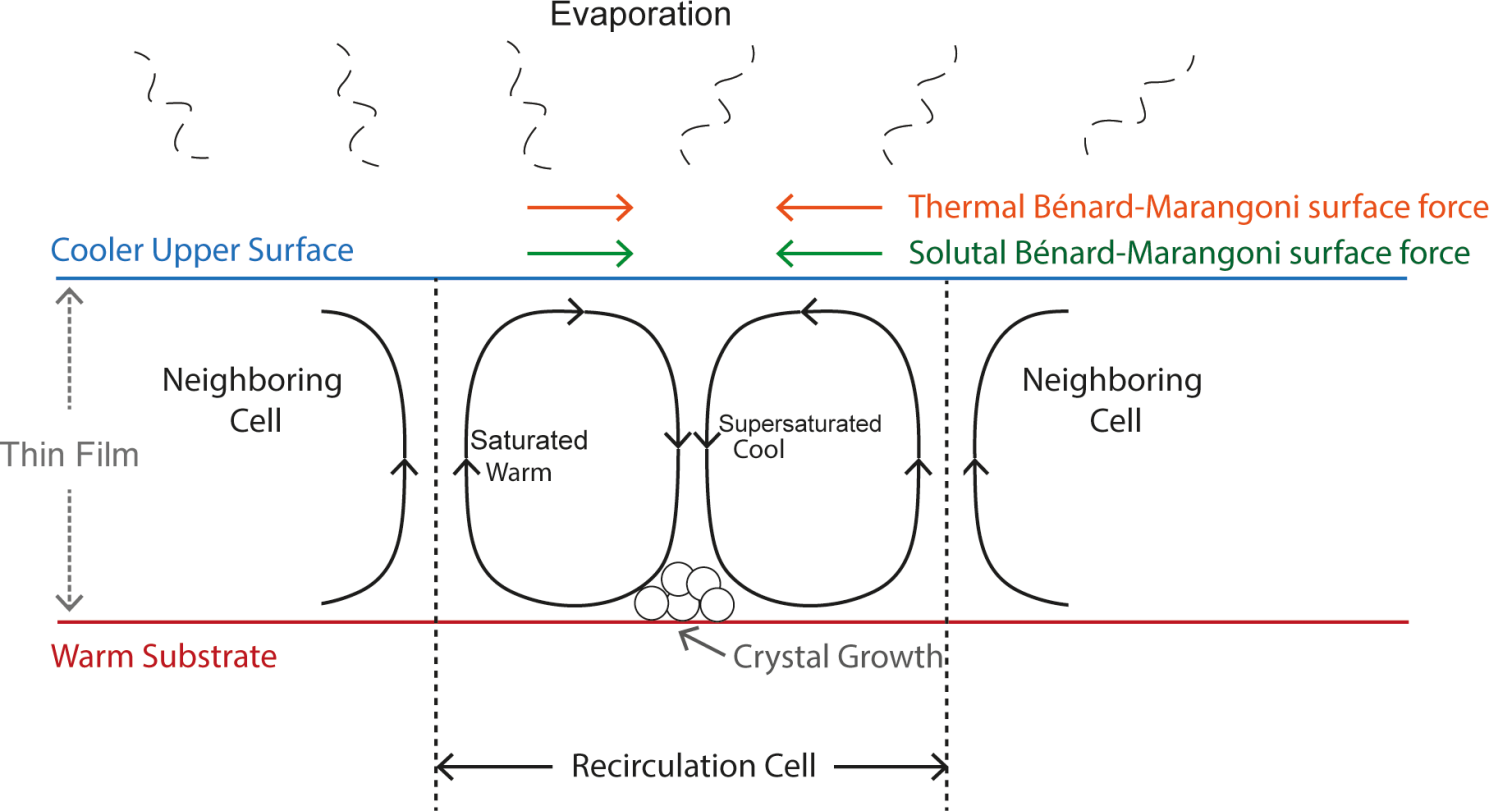
Schematic diagram of the convective cells forming in the films due to the development of instabilities.

Figure 8 shows a schematic diagram of the convective cells formed in the thin films, giving rise to the cell-like structures.

**Figure 9:**
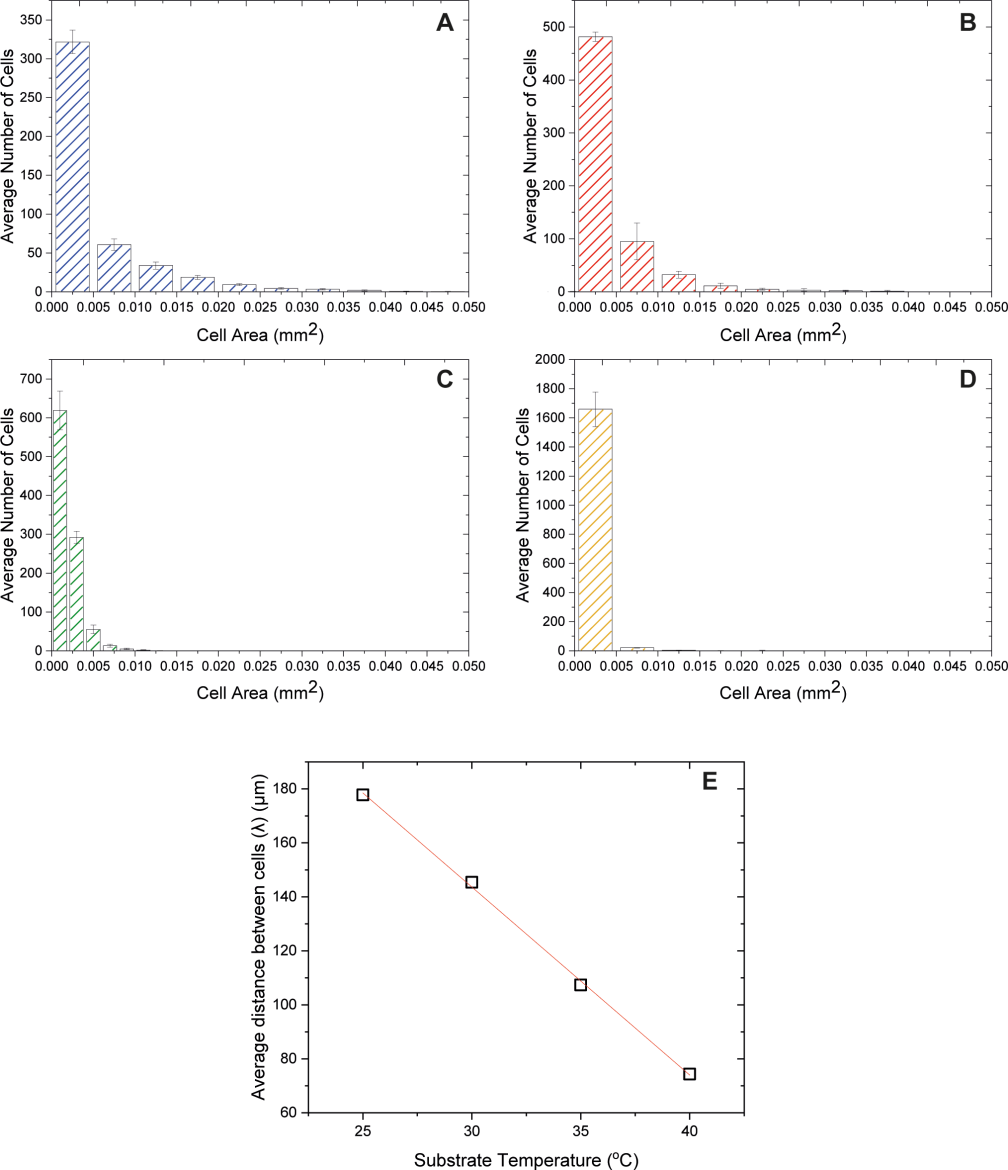
Average number and area distribution of cell-like structures observed in the crystalline area for droplets evaporating at (A) 25, (B) 30, (C) 35, and (D) 40 °C. (E) Average distance between cells (λ) with substrate temperature based on hexagonal geometry.

The area and distribution of cell-like structures were investigated for temperatures between 25 and 40 °C (Figure 9) via ImageJ (v1.53c) software. The number and area of the crystalline structures vary with temperature. It has been found that elevated temperatures give rise to a higher number of cell structures in the crystalline area. Structures with areas of 0.005 mm^2^ have been observed in the samples for all the examined temperatures. Nevertheless, at 25 and 30 °C, a number of larger structures exist in the central region with a maximum area of 0.025 and 0.020 mm^2^, respectively, leading to the conclusion that the base area of each cell decreases as the substrate temperature increases. This would be in agreement with stronger Bénard–Marangoni convection caused by the higher temperature gradient between the apex and the base of the drying drops, leading to a higher number of smaller convective cells. As a result of the cell formation, the salt diffusion within the drying droplets would be limited because fluid is trapped within each formed cell. At lower temperatures, on the other hand, evaporation is slower, enabling the spatial and temporal distribution of ions in the central zone through diffusion.

Convection gives rise to the formation of cells whose structure approximates a hexagonal geometry.^54^ In this study, the presence of impurities in the film during evaporation (because of protein aggregation) affects the cell formation, leading to irregular hexagonal cells. On the basis of the number of cell-like structures calculated via image analysis and considering the hexagonal-like structure of the cells, we calculate the average repetition distance of the cells (λ), often termed wavelength, as a function of the substrate temperature. Figure 9E shows the average repetition distance between cells (λ) as a function of the substrate temperature, suggesting that the average cell distance is inversely proportional to the substrate temperature. This decrease in the size and average distance between cells is consistent with a more intense thermal convection at higher substrate temperatures. A schematic of the average distance between cells (λ) along with a high-magnification image (10×) of the cell-like structures is presented in Figure S7.

### Effect of Temperature on Crystalline Growth

Temperature also affects the growing manner of the crystalline patterns. Dendrite nucleation is initiated with the formation of one or two crystal nuclei, which grow as a single crystal in an almost circular pattern, in the central region of the droplet (Figure S8). Crystallization is completed when the crystalline area reaches the gel zone. Conversely, for cell-like structures, multiple nucleation points appear simultaneously in the central region.

The existence of multiple nucleation points at higher temperatures is possible for two reasons. Firstly, the higher evaporation rate at higher substrate temperatures leads to the possibility of multiple nucleation sites, which would cause a more random nucleation process. Secondly, the number of crystal nuclei will be influenced by the spatial region over which circulation is restricted. Because of the regularity of the nucleation sites, we believe that the latter is the main reason for the existence of multiple nucleation sites at higher substrate temperatures. In the case of dendritic growth, one or two nuclei form, which grow and propagate within the central crystalline zone with no spatial restrictions. As a result, crystallization extends from the formed nucleation site, covering the crystalline zone. However, in the case of cell-like structures forming at higher substrate temperatures, when the thermal Bénard–Marangoni convection is stronger, the number of convective cells forming in the central crystalline zone increases. In this case, fluid is trapped in each convective cell and each cell does not share fluid with neighboring cells. At the latter evaporation stages, when solutal Bénard–Marangoni convection dominates, each convective cell may act as an individual nucleation site. This would mean that the number of crystal nuclei is related to the number of convective cells forming.

For droplets evaporating at 25 and 30 °C, crystals nucleate as circular individual spots in the central region of the drying drop, merging a few seconds after nucleation and propagating toward the periphery of the drop (Figures S9 and S10). For the highest examined temperatures of 35 and 40 °C, on the other hand, an arc-like crystal growth characterizes the onset of crystallization (Figures S11 and S12).

## Conclusions

The drying of biological fluid droplets gives rise to distinct desiccation patterns. The conditions under which drying takes place, such as temperature and relative humidity, affect the drying process and can lead to distinct variations in the dried deposits. Therefore, it is important to understand how these conditions affect the evaporative dynamics and the final deposit formation. In this work, we focus on the effect of substrate temperature on the crystalline pattern formation in dried deposits of FBS droplets. We examine substrate temperatures between 20 and 40 °C, as these are the most relevant for diagnostic applications. We estimate the forces acting at the onset of drying, finding that hydrodynamic fluid forces dominate.

Examining the desiccation deposits and focusing on the central crystalline zone, we find that the ratio of the crystalline area over the entire deposit area (*A*_c_/*A*_total_) decreases with substrate temperature. A transition in the morphology of the crystals is observed, from dendrites at 20 °C to cell-like structures for substrate temperatures between 25 and 40 °C. We believe that this transition occurs because of the interplay between thermal and solutal Bénard–Marangoni convection in the drying droplets, leading to the development of convective instabilities. In an attempt to explain the change in the morphology of the crystalline structures, we calculate the thermal and solutal Bénard–Marangoni numbers at the end of the gelation stage. Thermal effects are negligible throughout drying in the case of dendritic crystalline structures forming on substrates of 20 °C. However, in the case of cell-like structures, both thermal and solutal convective effects act within the drying drops. Thermal convective effects dominate during the early drying stages, leading to the development of instabilities and the formation of convective cells. As evaporation proceeds, the solutal effects dominate, maintaining the circulation and leading to crystallization within the formed cells. These findings support our hypothesis that the formation of cell-like structures results from the interplay between thermal and solutal effects in the drying droplets. In the case of dendrites, the crystalline zone is found to be significantly thicker compared to the case of cell-like structures. For cell-like structures, image analysis has shown that the number of cells increases with increasing substrate temperature, whereas the area occupied by each cell-like structure decreases. The average distance between cells (λ) has been calculated and was found to be inversely proportional to the substrate temperature. Temperature also affects the nucleation and growing manner of the crystals from one or two circular nuclei at 20 °C, to multiple circular nuclei that grow into crystals and merge at 25 and 30 °C, to an arc-like growth at higher temperatures.

The findings of this work could contribute to the understanding of the impact of environmental conditions on the desiccation patterns of biological fluid droplets as a tool for disease diagnosis. Investigation of FBS deposits at a larger range of temperatures may be required for a better understanding of the mechanisms affecting the final desiccation patterns; however, this was beyond the scope of this study.

## Supporting Information Available

Additional experimental information, calculations and values of parameters used for calculations, and figures (PDF)

## Abbreviations

BSA: bovine serum albumin
CCR: constant contact radius
FBS: fetal bovine serum

## References

[ref1] Deegan R. D.; Bakajin O.; Dupont T. F.; Huber G.; Nagel S. R.; Witten T. A. Capillary Flow as the Cause of Ring Stains from Dried Liquid Drops. Nature 1997, 389 (23), 827–829. 10.1038/39827.

[ref2] Xu X.; Luo J. Marangoni Flow in an Evaporating Water Droplet. Appl. Phys. Lett. 2007, 91, 124102 10.1063/1.2789402.

[ref3] Semenov S.; Starov V. M.; Rubio R. G.; Agogo H.; Velarde M. G. Evaporation of Sessile Water Droplets: Universal Behaviour in Presence of Contact Angle Hysteresis. Colloids Surfaces A Physicochem. Eng. Asp. 2011, 391 (1–3), 135–144. 10.1016/j.colsurfa.2011.07.013.

[ref4] Misyura S. Y. Evaporation of a Sessile Water Drop and a Drop of Aqueous Salt Solution. Sci. Rep. 2017, 7, 14759. 29116129 10.1038/s41598-017-15175-1 PMC5676712

[ref5] Christy J. R. E.; Hamamoto Y.; Sefiane K. Flow Transition within an Evaporating Binary Mixture Sessile Drop. Phys. Rev. Lett. 2011, 106, 205701. 21668243 10.1103/PhysRevLett.106.205701

[ref6] Sefiane K.; David S.; Shanahan M. E. R. Wetting and Evaporation of Binary Mixture Drops. J. Phys. Chem. B 2008, 112 (36), 11317–11323. 10.1021/jp8030418. 18707163

[ref7] Liu W.; Midya J.; Kappl M.; Butt H. J.; Nikoubashman A. Segregation in Drying Binary Colloidal Droplets. ACS Nano 2019, 13 (5), 4972–4979. 10.1021/acsnano.9b00459. 30897326 PMC6727607

[ref8] Maki K. L.; Kumar S. Fast Evaporation of Spreading Droplets of Colloidal Suspensions. Langmuir 2011, 27 (18), 11347–11363. 10.1021/la202088s. 21834573

[ref9] Patil N. D.; Bange P. G.; Bhardwaj R.; Sharma A. Effects of Substrate Heating and Wettability on Evaporation Dynamics and Deposition Patterns for a Sessile Water Droplet Containing Colloidal Particles. Langmuir 2016, 32 (45), 11958–11972. 10.1021/acs.langmuir.6b02769. 27759960

[ref10] Sung P. F.; Wang L.; Harris M. T. Deposition of Colloidal Particles during the Evaporation of Sessile Drops: Dilute Colloidal Dispersions. Int. J. Chem. Eng. 2019, 2019, 1–12. 10.1155/2019/7954965.

[ref11] Choudhury M. D.; Dutta T.; Tarafdar S. Pattern Formation in Droplets of Starch Gels Containing NaCl Dried on Different Surfaces. Colloids Surfaces A Physicochem. Eng. Asp. 2013, 432, 110–118. 10.1016/j.colsurfa.2013.04.064.

[ref12] Kaya D.; Belyi V. A.; Muthukumar M. Pattern Formation in Drying Droplets of Polyelectrolyte and Salt. J. Chem. Phys. 2010, 133, 114905 10.1063/1.3493687. 20866155

[ref13] Takhistov P.; Chang H. Complex Stain Morphologies. Ind. Eng. Chem. Res. 2002, 41, 6256–6269. 10.1021/ie010788+.

[ref14] Yakhno T. Salt-Induced Protein Phase Transitions in Drying Drops. J. Colloid Interface Sci. 2008, 318, 225–230. 10.1016/j.jcis.2007.10.020. 18001759

[ref15] Sobac B.; Brutin D. Desiccation of a Sessile Drop of Blood: Cracks, Folds Formation and Delamination. Colloids Surfaces A Physicochem. Eng. Asp. 2014, 448 (1), 34–44. 10.1016/j.colsurfa.2014.01.076.

[ref16] Chen R.; Zhang L.; Zang D.; Shen W. Blood Drop Patterns : Formation and Applications. Adv. Colloid Interface Sci. 2016, 231, 1–14. 10.1016/j.cis.2016.01.008. 26988066

[ref17] Annarelli C. C.; Fornazero J.; Bert J.; Colombani J. Crack Patterns in Drying Protein Solution Drops. Eur. Phys. J. E 2001, 603, 599–603.

[ref18] Gorr H. M.; Zueger J. M.; McAdams D. R.; Barnard J. A. Salt-Induced Pattern Formation in Evaporating Droplets of Lysozyme Solutions. Colloids Surfaces B Biointerfaces 2013, 103, 59–66. 10.1016/j.colsurfb.2012.09.043. 23201720

[ref19] Pauchard L.; Parisse F.; Allain C. Influence of Salt Content on Crack Patterns Formed through Colloidal Suspension Desiccation. Phys. Rev. E - Stat. Physics, Plasmas, Fluids, Relat. Interdiscip. Top. 1999, 59 (3), 3737–3740. 10.1103/PhysRevE.59.3737.

[ref20] Tarasevich Y. Y.; Ayupova A. K. Effect of Diffusion on the Separation of Components in a Biological Fluid upon Wedge-Shaped Dehydration. Technol. Phys. 2003, 48 (5), 535–540. 10.1134/1.1576463.

[ref21] Killeen A. A.; Ossina N.; Mcglennen R. C.; Minnerath S.; Borgos J. Protein Self-Organization Patterns in Dried Serum Reveal Changes in B-Cell Disorders. Mol. Diagn. Ther. 2006, 10 (6), 371–380. 10.1007/BF03256214. 17154654

[ref22] Laan N.; De Bruin K. G.; Slenter D.; Wilhelm J.; Jermy M.; Bonn D. Bloodstain Pattern Analysis: Implementation of a Fluid Dynamic Model for Position Determination of Victims. Sci. Rep. 2015, 5 (June), 1–8. 10.1038/srep11461. PMC4476491 26099070

[ref23] Brutin D.; Sobac B.; Loquet B.; Sampol J. Pattern Formation in Drying Drops of Blood. J. Fluid Mech. 2011, 667, 85–95. 10.1017/S0022112010005070.

[ref24] Yakhno T. A.; Yakhno V. G.; Sanin A. G.; Sanina O. A.; Pelyushenko A. S.; Egorova N. A.; Terentiev I. G.; Smetanina S. V.; Korochkina O. V.; Yashukova E. V. The Informative-Capacity Phenomenon of Drying Drops. IEEE Eng. Med. Biol. Mag. 2005, 24 (2), 96–104. 10.1109/MEMB.2005.1411354. 15825851

[ref25] Smith F. R.; Nicloux C.; Brutin D. A New Forensic Tool to Date Human Blood Pools. Sci. Rep. 2020, 10 (1), 1–12. 10.1038/s41598-020-65465-4. 32451419 PMC7248111

[ref26] Yakhno T. A.; Yakhno V. G. Structural Evolution of Drying Drops of Biological Fluids. Technol. Phys. 2009, 54 (8), 1219–1227. 10.1134/S1063784209080210.

[ref27] Shabalin V. N.; Shatokhina S. N. Diagnostic Markers in the Structures of Human Biological Liquids. Singapore Med. J. 2007, 48 (5), 440–446. 17453103

[ref28] Yakhno T. A.; Sedova O. A.; Sanin A. G.; Pelyushenko A. S. On the Existence of Regular Structures in Liquid Human Blood Serum (Plasma) and Phase Transitions in the Course of Its Drying. Technol. Phys. 2003, 48 (4), 399–403. 10.1134/1.1568479.

[ref29] Sobac B.; Brutin D. Desiccation of a Sessile Drop of Blood : Cracks, Folds Formation and Delamination. Colloids Surfaces A Physicochem. Eng. Asp. 2014, 448, 34–44. 10.1016/j.colsurfa.2014.01.076.

[ref30] Roy B.; Choudhuri M. D.; Dutta T.; Tarafdar S. Multi-Scale Patterns Formed by Sodium Sulphate in a Drying Droplet of Gelatin. Appl. Surf. Sci. 2015, 357, 1000–1006. 10.1016/j.apsusc.2015.09.085.

[ref31] Annarelli C. C.; Reyes L.; Fornazero J.; Bert J. Ion and Molecular Recognition Effects on the Crystallisation of Bovine Serum Albumin - Salt Mixtures. Cryst. Eng. 2000, 3, 173–194. 10.1016/S1463-0184(00)00038-1.

[ref32] Pathak B.; Christy J.; Sefiane K.; Gozuacik D. Complex Pattern Formation in Solutions of Protein and Mixed Salts Using Dehydrating Sessile Droplets. Langmuir 2020, 36 (33), 9728–9737. 10.1021/acs.langmuir.0c01122. 32787115

[ref33] Tarasevich Y. Y.; Pravoslavnova D. M. Drying of a Multicomponent Solution Drop on a Solid Substrate : Qualitative Analysis. Technol. Phys. 2007, 52 (2), 159–163. 10.1134/S106378420702003X.

[ref34] Chen R.; Zhang L.; He H.; Shen W. Desiccation Patterns of Plasma Sessile Drops. ACS Sensors 2019, 4 (6), 1701–1709. 10.1021/acssensors.9b00618. 31099244

[ref35] Esmonde-white K. A.; Esmonde-white F. W. L.; Morris M. D.; Blake J.; Arbor A.; Arbor A. Characterization of Biofluids Prepared by Sessile Drop Formation. Analyst. 2015, 139 (11), 2734–2741. 10.1039/c3an02175k. PMC4077870 24757707

[ref36] Brutin D.; Sobac B. Influence of Substrate Nature on the Evaporation of a Sessile Drop of Blood. J. Heat Trasnfer 2012, 134 (June 2012), 1–7. 10.1115/1.4006033.

[ref37] Zeid W. B.; Brutin D. Influence of Relative Humidity on Spreading, Pattern Formation and Adhesion of a Drying Drop of Whole Blood. Colloids Surf., A 2013, 430, 1–7. 10.1016/j.colsurfa.2013.03.019.

[ref38] Carreón Y. J. P.; Ríos-Ramírez M.; Vázquez-Vergara P.; Salinas-Almaguer S.; Cipriano-Urbano I.; Briones-Aranda A.; Díaz-Hernández O.; Escalera Santos G. J.; González-Gutiérrez J. Effects of Substrate Temperature on Patterns Produced by Dried Droplets of Proteins. Colloids Surf., B 2021, 203 (April), 1–10. 10.1016/j.colsurfb.2021.111763. 33865091

[ref39] Krishnan A.; Siedlecki C. A.; Vogler E. A. Mixology of Protein Solutions and the Vroman Effect. Langmuir 2004, 20 (12), 5071–5078. 10.1021/la036218r. 15984270

[ref40] Chen R. Understanding Desiccation Patterns of Blood Sessile Drops Sessile Drops. J. Mater. Chem. B 2017, 5 (October), 8991–8998. 32264126 10.1039/c7tb02290e

[ref41] Shen C.-H. Diagnostic Molecular Biology, 1st ed.; Elsevier: 2019.

[ref42] Kaneko J. J.; Harvey J. W.; Bruss M. L. Clinical Biochemistry of Domestic Animals, 6th ed.; Academic Press: 2008.

[ref43] Roth C. M.; Neal B. L.; Lenhoff A. M. Van Der Waals Interactions Involving Proteins. Biophys. J. 1996, 70, 977–987. 10.1016/S0006-3495(96)79641-8. 8789115 PMC1224998

[ref44] Sett A.; Ayushman M.; Desgupta S.; Dasgupta S. Analysis of the Distinct Pattern Formation of Globular Proteins in the Presence of Micro- and Nanoparticles. J. Phys. Chem. B 2018, 122 (38), 8972–8984. 10.1021/acs.jpcb.8b05325. 30185036

[ref45] Rathaur V. S.; Kumar S.; Panigrahi P. K.; Panda S. Investigating the Effect of Antibody-Antigen Reactions on the Internal Convection in a Sessile Droplet via Microparticle Image Velocimetry and DLVO Analysis. Langmuir 2020, 36 (30), 8826–8838. 10.1021/acs.langmuir.0c01162. 32628853

[ref46] Israelachvili J. N. Intermolecular and Surface Forces, 3rd ed.; 2011; Vol. 59.

[ref47] Leckband D.; Israelachvili J. Quarterly Reviews of Biophysics; Cambridge University Press: 2001; Vol. 34. 10.1017/s0033583501003687 11771120

[ref48] Bassou N.; Rharbi Y. Role of Bénard-Marangoni Instabilities during Solvent Evaporation in Polymer Surface Corrugations. Langmuir 2009, 25 (1), 624–632. 10.1021/la802979a. 19032111

[ref49] Bestehorn M. Phase and Amplitude Instabilities for Bénard-Marangoni Convection in Fluid Layers with Large Aspect Ratio. Phys. Rev. E 1993, 48 (5), 3622–3634. 10.1103/PhysRevE.48.3622. 9961019

[ref50] Pearson J. R. A. On Convection Cells Induced by Surface Tension. J. Fluid Mech. 1958, 4 (5), 489–500. 10.1017/S0022112058000616.

[ref51] Huang J.; Ali N.; Quansah E.; Guo S.; Noutsias M.; Meyer-Zedler T.; Bocklitz T.; Popp J.; Neugebauer U.; Ramoji A. Vibrational Spectroscopic Investigation of Blood Plasma and Serum by Drop Coating Deposition for Clinical Application. Int. J. Mol. Sci. 2021, 22 (4), 1–19. 10.3390/ijms22042191. PMC7926873 33671841

[ref52] Chen R.; Zhang L.; He H.; Shen W. Desiccation Patterns of Plasma Sessile Drops. ACS Sensors 2019, 4 (6), 1701–1709. 10.1021/acssensors.9b00618. 31099244

[ref53] Buzoverya M. E.; Shcherbak Y. P.; Shishpor I. V. Experimental Investigation of the Serum Albumin Fascia Microstructure. Technol. Phys. 2012, 57 (9), 1270–1276. 10.1134/S1063784212090071.

[ref54] Maroto J. A.; Pérez-Mũuzuri V.; Romero-Cano M. S. Introductory Analysis of Bénard-Marangoni Convection. Eur. J. Phys. 2007, 28 (2), 311–320.

