# Supplementary material for: Transition from Dendritic to Cell-like Crystalline Structures in Drying Droplets of Fetal Bovine Serum under the Influence of Temperature
Source: Langmuir. 2022 Mar 31;38(14):4321–31. doi: 10.1021/acs.langmuir.2c00019 (PMC9009182; doi:10.1021/acs.langmuir.2c00019)
Supplement: Supplementary file 1 — la2c00019_si_001.pdf [file la2c00019_si_001.pdf]

Transition from Dendritic to Cell-like Crystalline Structures in Drying Droplets of Fetal Bovine Serum Under the Influence of Temperature

Marina Efstratiou[1,2], John Christy[2], Daniel Bonn[3] and Khellil Sefiane[2]*

1. *Division of Pharmacy and Optometry, Faculty of Biology, Medicine and Health, The University of Manchester, Stopford Building, Oxford Road, Manchester M13 9PL*
2. *Institute of Multiscale Thermofluids, School of Engineering, The University of Edinburgh, King's Buildings, James Clerk Maxwell Building, Peter Guthrie Tait Road, King's Buildings, Edinburgh EH9 3FD*
3. *Institute of Physics, University of Amsterdam, Science Park 904, 1098XH Amsterdam, The Netherlands*

Corresponding Author: *E-mail: J.Christy@ed.ac.uk

Table of Contents

Experimental Setup S3

Hydrodynamic and Intermolecular Forces Acting within the Droplets at the Onset of Evaporation S4

Discussion on Calculated DLVO Forces S12

Limitations of DLVO theory S14

Gel zones in desiccation deposits of FBS droplets...S15

Times for the Onset of Crystallization S17

Values of Parameters used for the Calculation of Bénard-Marangoni numbers S18

Normalized Height with time for FBS Droplets Drying on Substrates of Different Temperatures S19

Calculated thermal Bénard-Marangoni numbers with time during drying S20

Cell-like Structures and cell wavelength (λ) S21

Effect of Temperature on Crystalline Growth S22

REFERENCES..S27

Experimental Setup

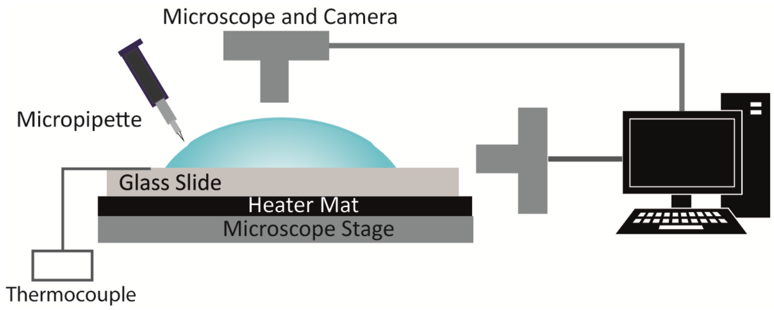

Figure S1: Schematic diagram of the experimental setup**.**

Hydrodynamic and Intermolecular Forces Acting within the Droplets at the Onset of Evaporation

In what follows, we attempt to estimate the order of magnitude of hydrodynamic fluid (capillary, drag) and intermolecular forces acting at the onset of evaporation in Fetal Bovine Serum (FBS) droplets drying on glass slides of different temperatures. The internal flows developing in the drying drops are affected by the interplay of multiple interaction forces including capillary and drag forces acting due to drying, as well as Van der Waals and electrostatic forces between the protein macromolecules, and between the macromolecules and the substrate.

Hydrodynamic Fluid Forces

Capillary Force. During evaporation of the droplets on the hydrophilic glass slides, the evaporation rate is higher at the contact line, giving rise to an outward flow. This flow carries the biomolecules toward the contact line, where they are deposited. As the biomolecules deposit on the contact line, they cause perturbations in the shape of the liquid near the air/liquid interface, giving rise to menisci. The overlap of the menisci around two macromolecules gives rise to a lateral capillary force, which affects the aggregation and packing of macromolecules at the periphery. This capillary force develops when molecules (or particles) are partially immersed in a liquid layer. In this case, the deformation of the liquid surface depends on the wetting properties of the molecule's surface. The capillary force is given by

$$F_C = 2\pi r\gamma cos\theta \qquad \text{(S.1)}$$

where r is the radius of a protein or an ion, γ is the surface tension of serum and θ is the contact angle between the substrate and the drying droplet[1,2].

Drag Force. Additionally, the internal flow imposes drag forces on macromolecules and ions. The drag force is expressed by:

$$F_D = 6\pi r \eta v \quad \text{(S.2)}$$

where η is the fluid viscosity and ν is the flow velocity due to evaporation.

Intermolecular Forces

The stability of colloidal solutions may be evaluated via the DLVO (Derjaguin, Landau, Verwey, and Overbeek) theory, which takes into account the attractive Van der Waals and repulsive electrostatic double layer intermolecular forces. Based on the attractive and electrostatic forces, the overall force acting on macromolecules may be calculated, at each separation distance[3].

Our solution is a complex system, consisting of various proteins. In order to enable the estimation of the interaction forces, we consider a simpler model of two proteins in an aqueous saline solution. FBS is composed of BSA and different types of globulins (alpha, beta and gamma). The type and concentration of proteins in FBS are given in Table 1. For the estimation of the interaction forces we consider that FBS consists of BSA and alpha globulins, which are the most abundant proteins in the mixture. For the calculations we consider a specific type of alpha-globulins, ceruloplasmin, which is used in diagnostic applications[4–6]. The molecular weights of BSA and ceruloplasmin are 66.5 kDa and 135 kDa, respectively. BSA and ceruloplasmin are considered as hard spheres with radii of 3.5 nm and 4.56 nm respectively. We calculate the total interaction forces between protein macromolecules (BSA-BSA, α globulin - α globulin and BSA-α globulin) for two temperatures: 25°C and 40°C. The interaction forces between the biomolecules and the substrate are also calculated.

Van der Waals forces. The Van der Waals attractive force for two spherical macromolecules of radii R_1 and R_2 respectively, at a separation distance D, is given by:

$$F_{VdWpp} = \frac{-A_{131}}{6D^2}\left(\frac{R1R2}{R1+R2}\right) \qquad \text{(S.3)}$$

where A_{131} is the Hamaker constant between the proteins in the medium, D is the minimum separation distance, and R_1 and R_2 are the radii of the macromolecules[7]. The separation distance was calculated considering the molecular weight and the concentration of proteins in the mixture. The separation distance differs for BSA-BSA, globulin-globulin and BSA-globulin interactions.

Additionally, the Van der Waals force between the proteins and the substrate is given by:

$$F_{VdWps} = \frac{-2\,A_{132}\,R^3}{3D^2(D+2R)^2} \qquad \text{(S.4)}$$

where A_{132} is the Hamaker constant between the proteins and the substrate.

Electrostatic Double Layer Forces. The electrostatic double layer protein-protein repulsion is affected by the ionic strength of the solution and is given by:

$$F_{EDLpp} = \kappa Z e^{-\kappa D}\left(\frac{R1R2}{R1+R2}\right) \qquad \text{(S.5)}$$

For the estimation of the electrostatic force, Z is given by:

$$Z = 64\,\pi\varepsilon_o\varepsilon\left(\frac{\mathrm{k_B T}}{\mathrm{e}}\right)^2 tanh^2\left(\frac{ze\psi_o}{4k_BT}\right) \qquad \text{(S.6)}$$

where z is the electrolyte valence ($z = 1$ for monovalent ions) and ψ_o is the zeta potential of the solution[7]. In this work, it was assumed that the proteins have similar zeta potentials at each substrate temperature (~ -20 mV at 25ºC and ~-14mV at 40ºC).

The Debye Length, k^{-1} is a measure of the net electrostatic effect of a charged macromolecule in a solution and the extent at which its electrostatic effect persists[8]. Debye Length is calculated by:

$$k^{-1} = \sqrt{\frac{\varepsilon_0 \varepsilon k_B T}{2 N_A e^2 I}} \quad \text{(S.7)}$$

where ε_0 is the permittivity of vacuum (8.854 x 10^{-12} C^2/ N m^2), ε is the relative dielectric constant of serum, k_B is the Boltzmann constant (1.381 x 10^{-23} J/K); T is the absolute temperature (in Kelvin) during the experimental procedure, N_A is the Avogadro number ($6.02214076 \times 10^{23}$ mol^{-1}), e is the elementary charge (1.602 x 10^{-19}C) and I is the ionic strength of the solution[7]. We use the values for the dielectric constant of plasma, as an approximation for those of serum. The values of the dielectric constants are given in Table S1.

Table S1: Dielectric constant of plasma with temperature [9]**.**

Temperature (°C)	Relative Dielectric Constant
20	67.107
25	66.07
30	65.259
35	64.622
40	63.518

The ionic strength is given by:

$$I = \frac{1}{2}\sum_{i} c_i z_i^2 \qquad \text{(S.8)}$$

where c_i is the molar concentration of an ion i and z_i is the ion's valence. Based on the FBS composition used in this study, the ionic strength of the solution at the onset of drying is ~0.14M. For the estimation of the interaction forces we have considered a monovalent salt (NaCl, which is the most abundant salt in blood serum).

The protein-substrate repulsion is given by:

$$F_{EDLps} = \kappa R Z e^{-\kappa D} \qquad \text{(S.9)}$$

In this case, Z is expressed as:

$$Z = 64\,\pi\varepsilon_o\varepsilon\left(\frac{\mathrm{k_B T}}{\mathrm{e}}\right)^2 tanh\left(\frac{ze\psi_o}{4k_BT}\right)\,tanh\left(\frac{ze\psi_s}{4k_BT}\right) \qquad \text{(S.10)}$$

where ψ_s is the zeta potential of the substrate (~ -62 mV).

The sum of the electrostatic repulsive and the Van der Waals attractive forces gives the total DLVO interaction force of the system at a specific separation distance[3]:

$$F_{DLVO=}\,F_{VdW} + F_{EDL} \qquad \text{(S.11)}$$

The values of the parameters used for the hydrodynamic fluid and intermolecular force calculations are given in Table S2.

Table S2: Parameters used for the calculation of forces in an aqueous saline solution consisting of two types of proteins, drying on a glass slide.

**Symbol**	**Parameter**	**Value**	**Units**
γ (25ºC)	Surface tension of Serum at 25ºC	0.057	N/m
γ (40ºC)	Surface tension of Serum at 25ºC	0.051	N/m
η (25ºC)	Dynamic Viscosity of Serum at 25ºC[10]	0.0017	Pa·s
η (40ºC)	Dynamic Viscosity of Serum at 40ºC[10]	0.0012	Pa·s
R_{BSA}	BSA radius[11]	3.5	nm
$R_{\alpha\text{-globulin}}$	α-globulin radius [11]	4.56	nm
R_{ion}	Ion radius	0.227	nm
A_{131} (25ºC)	Hamaker constant between proteins at 25ºC[12–15]	1 x 10^{-20}	J
A_{131} (40ºC)	Hamaker constant between proteins at 40ºC[12–15]	2 x 10^{-20}	J
A_{132}	Hamaker constant between proteins and glass substrate[1,15]	3 x 10^{-20}	J

Table S3 shows the name and explanation of the calculated forces.

Table S3: Table of nomenclature for calculated forces.

Name of Force	Meaning
F_C	Capillary forces
F_D	Drag force
F_{VdW}	Van der Waals intermolecular forces
F_{EDL}	Electrostatic intermolecular forces

For a FBS droplet drying on a hydrophilic glass slide of 25ºC, with an initial contact angle of 35º, the forces acting on biomolecules and ions at the onset of desiccation, are given in Table S4. Subscripts BSA, αglob and ion indicate forces acting on BSA macromolecules, α globulin macromolecules and ions, respectively. Pairs of BSA-BSA, BSA-sub, αglob-aglob, αglob-sub and BSA-αglob indicate the intermolecular BSA-BSA, BSA-substrate, α globulin-α globulin, α globulin-substrate and BSA-α globulin interactions respectively.

Table S4: Order of magnitude of the forces acting on the different types of protein macromolecules (BSA and α-globulin) and ions at the onset of evaporation, when the substrate temperature is 25°C.

**Type of Force**	**Magnitude (N)**
$F_{C\text{-}BSA}$	O(10^{-9})
$F_{C\text{-}glob}$	O(10^{-9})
$F_{C\text{-}ion}$	O(10^{-11})
$F_{D\text{-}BSA}$	O(10^{-17})
$F_{D\text{-}glob}$	O(10^{-17})
$F_{D\text{-}ion}$	O(10^{-18})
$F_{VdW\ BSA\text{-}BSA}$	O(10^{-14})
$F_{VdW\ BSA\text{-}sub}$	O(10^{-14})
$F_{EDL\ BSA\text{-}BSA}$	O(10^{-22})
$F_{EDL\ BSA\text{-}sub}$	O(10^{-21})
$F_{VdW\ glob\text{-}glob}$	O(10^{-14})
$F_{VdW\ glob\text{-}sub}$	O(10^{-14})
$F_{EDL\ glob\text{-}glob}$	O(10^{-26})
$F_{EDL\ glob\text{-}sub}$	O(10^{-25})
$F_{VdW\ BSA\text{-}glob}$	O(10^{-14})
$F_{EDL\ BSA\text{-}glob}$	O(10^{-16})

Discussion on Calculated DLVO Forces

Capillary forces are found to be the dominant forces acting on protein macromolecules (O (10^{-9} N) and they are of the same order of magnitude for droplets drying at 25ºC and 40ºC. Drag forces acting on proteins at 25ºC (O (10^{-17} N)) are one order of magnitude lower compared to those acting at 40ºC (O (10^{-16} N)). Drag forces acting on ions are of the order of (O (10^{-18} N)) for all of the examined substrate temperatures.

Upon transfer to the contact line, and while the droplet is still liquid, the biomolecules interact with each other and with the substrate through attractive Van der Waals and repulsive electrostatic forces, according to the DLVO (Derjaguin, Landau, Verwey, and Overbeek) theory. The physiological salt concentration in the mixture influences the DLVO forces due to electrostatic screening effects. This causes the Van der Waals protein-protein and protein-substrate attraction to be orders of magnitude higher than the electrostatic repulsion between protein macromolecules or between the proteins and the substrate for both 25ºC and 40ºC. This suggests that, at the molecular level, intermolecular attractive protein-protein and protein-substrate forces dominate over electrostatic forces in all cases, enhancing both the aggregation of proteins and their adhesion to the substrate, promoting contact line pinning. Additionally, the findings suggest that the attraction between macromolecules increases with increasing substrate temperature, whereas the protein-substrate interactions decrease with increasing substrate temperature from 25ºC to 40ºC.

The estimated Van der Waals forces between BSA macromolecules at the onset of drying are of the order of O (10^{-14} N) for 25ºC and 40ºC.For BSA-substrate interactions, the attractive forces at 25ºC (O (10^{-14} N)) are one order of magnitude higher compared to those at 40ºC (O (10^{-15} N)). For α globulins, interestingly, the Van der Waals forces between the biomolecules increase by an order

of magnitude from 25ºC (O (10^{-15} N)) to 40ºC (O (10^{-14} N)), however the interactions between the macromolecules and the substrate drop by an order of magnitude from 25ºC (O (10^{-14} N)), to 40ºC(O (10^{-15} N)). The attractive forces between BSA macromolecules are higher compared to those acting between α-globulins for both temperatures.

It is noteworthy that as drying proceeds, water evaporation leads to decreasing separation distance *D* between macromolecules, affecting intermolecular interactions. The Van der Waals attractive force increases as the separation distance decreases. The electrostatic repulsive force is also affected by water evaporation. Besides decreasing separation distance, water evaporation leads to increasing salt concentration within the drying drops. The increasing of the ionic strength during drying, affects the electrostatic repulsion between the macromolecules. It is expected that as the ionic strength increases, the Debye length decreases, weakening further the repulsive forces between proteins, thus promoting aggregation. The change in the net DLVO forces will be a result of the interplay between the changing separation distance and ionic strength of the solution.

Limitations of DLVO theory

It is important to note the limitations of DLVO theory in the case of protein-salt mixtures. Proteins are complex macromolecules with various dynamic conformations and similarly to colloids, they are charged. Nevertheless, the charge distribution on the surface of proteins is not homogeneous as in the case of colloids. Because of the heterogeneous surface charge, the interaction potential for proteins includes both DLVO and non-DLVO forces (such as hydration forces and hydrophobic interactions)[16]. Additionally, DLVO theory treats ion-colloid interactions as purely electrostatic and does not take into account ion specific Hofmeister effects and dispersion forces. This is particularly important at biological salt concentrations and above, where non-electrostatic (NES) ion-specific forces take place leading to ion-specific hydration[17]. Finally, the applicability of DLVO theory is restricted to monovalent and divalent ions. Therefore, for the complete understanding of the occurring phenomena, the aforementioned interactions should be taken into account[17].

Gel zones in desiccation deposits of FBS droplets

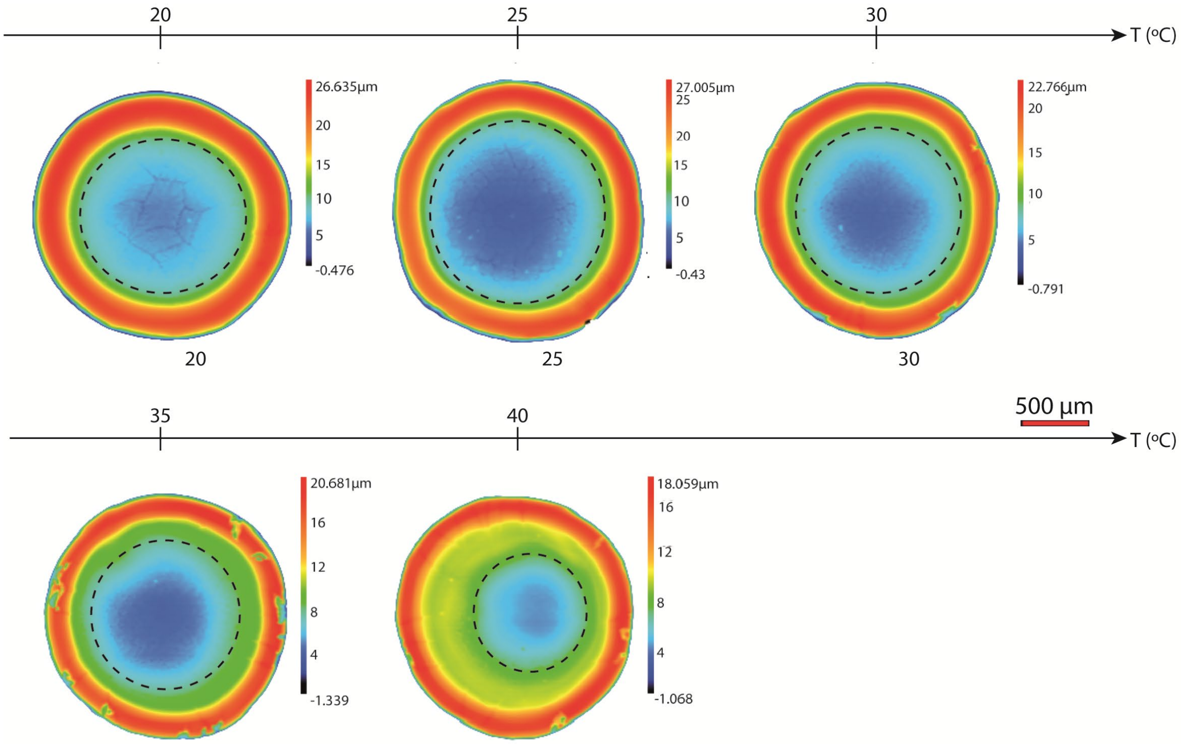

Figure S2*:* Images acquired via Keyence VK-X1000, showing the different zones in the final desiccated drops, where red is the ring, yellow and green the gel with the protein structures and blue the zone of crystalline structures (shown in Figure 3) respectively. It should be noted that each zone has a different average height, depending on the substrate temperature during drying. Color maps show the height of each zone in the deposits for different substrate temperature. The images show an increased protein area with an accompanying reduction of the crystalline area in the final dried deposits as the substrate temperature increases from 20ºC to 40ºC.

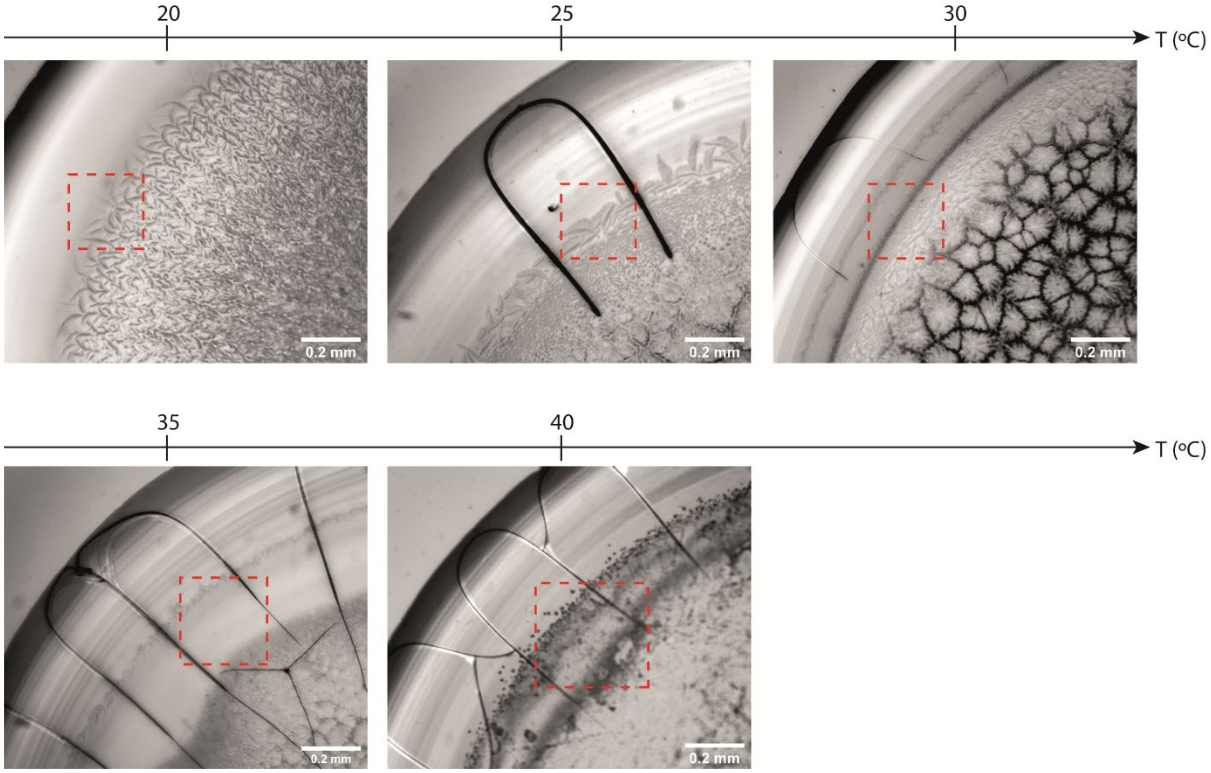

Figure S3: Gel regions in the final deposits of FBS drops desiccated at different substrate temperatures. The gel region is more profound at higher substrate temperatures (magnification: 10x). The images were acquired 24 hours after the completion of the experiments.

Times for the Onset of Crystallization

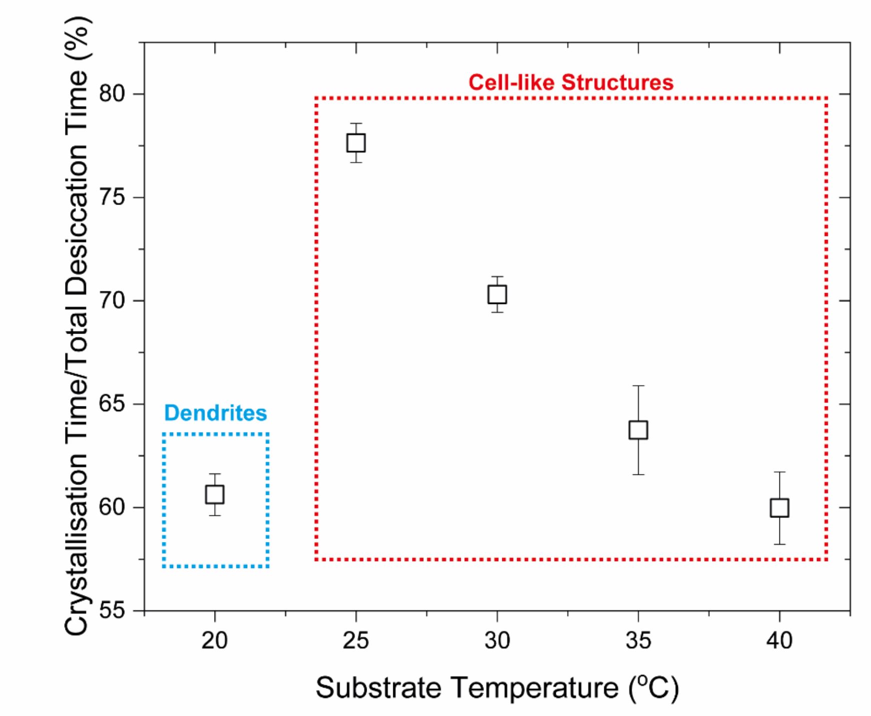

Figure S4: The time for the onset of crystallization differs for FBS droplets evaporating at different substrate temperatures. For droplets drying at 20ºC, crystallization occurs at ~60% of the drop lifetime, giving rise to dendritic patterns. However, for droplets evaporating at 25ºC the onset of crystallization is delayed and the time at which crystallization commences decreases between 25ºC- 40ºC. For these temperatures, cell-like patterns are observed in the central region of the drops.

Values of Parameters used for the Calculation of Bénard-Marangoni numbers

Table S5: Values of parameters used for the calculation of solutal and thermal Bénard-Marangoni numbers.

**Symbol**	**Parameter**	**Value**	**Units**
$\frac{\partial \gamma}{\partial C}$	Change in surface tension with concentration[18]	0.00162	$\frac{\frac{N}{m}}{\frac{mol}{L}}$
ΔC	Concentration difference[19] (C_{ssat} – C_{sat})	~3.1 (8.3-5.2)	$\frac{mol}{L}$
η (25°C)	Dynamic Viscosity of Serum at 20°C[10]	0.00195	Pa·s
η (25°C)	Dynamic Viscosity of Serum at 25°C[10]	0.0017	Pa·s
η (30°C)	Dynamic Viscosity of Serum at 30°C[10]	0.0015	Pa·s
η (35°C)	Dynamic Viscosity of Serum at 25°C[10]	0.0013	Pa·s
η (40°C)	Dynamic Viscosity of Serum at 40°C[10]	0.0012	Pa·s
D	Diffusion Coefficient[20]	$1.5\ x10^{-9}$	m^2/s
$\frac{\partial \gamma}{\partial T}$	Change in surface tension with temperature[20]	$3.68\ x10^{-4}$	$\frac{\frac{N}{m}}{K}$
α	Thermal Diffusivity[21]	$1.1\ x10^{-7}$	m^2/s

Normalized Height with time for FBS Droplets Drying on Substrates of Different Temperatures

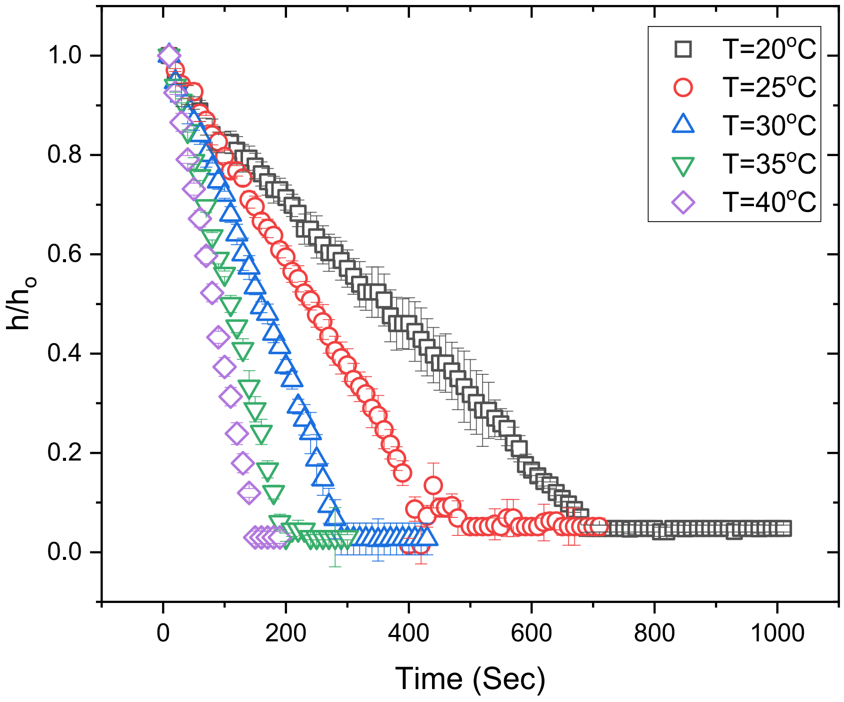

Figure S5: Normalized height of the drying drops with time during the evaporation process, for FBS droplets drying on glass slides of different temperatures. The height has been normalized based on the initial height of the drops at the onset of drying.

Calculated thermal Bénard-Marangoni numbers with time during drying

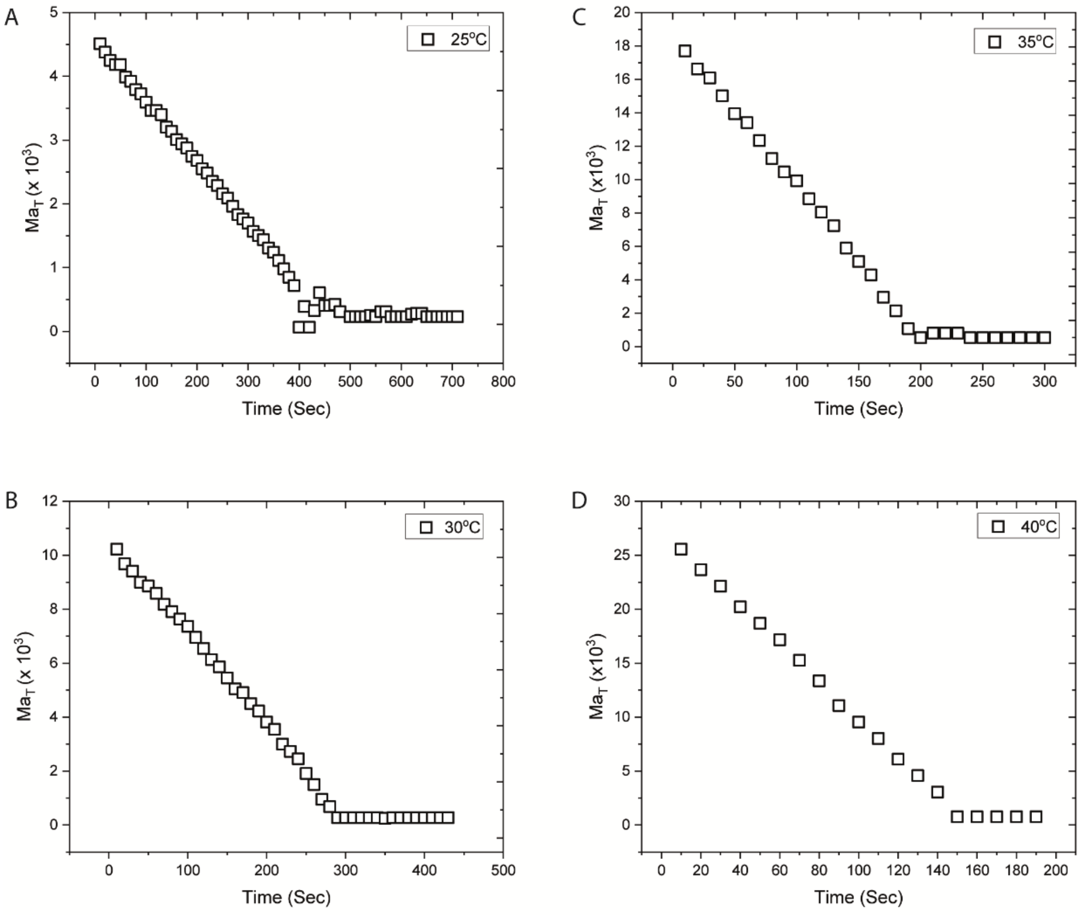

Figure S6: Estimated thermal Bénard-Marangoni numbers (Ma_T) as a function of time for FBS droplets drying on substrates of A) 25ºC, B) 30ºC, C) 35ºC and D) 40ºC.

Cell-like Structures and cell wavelength (λ)

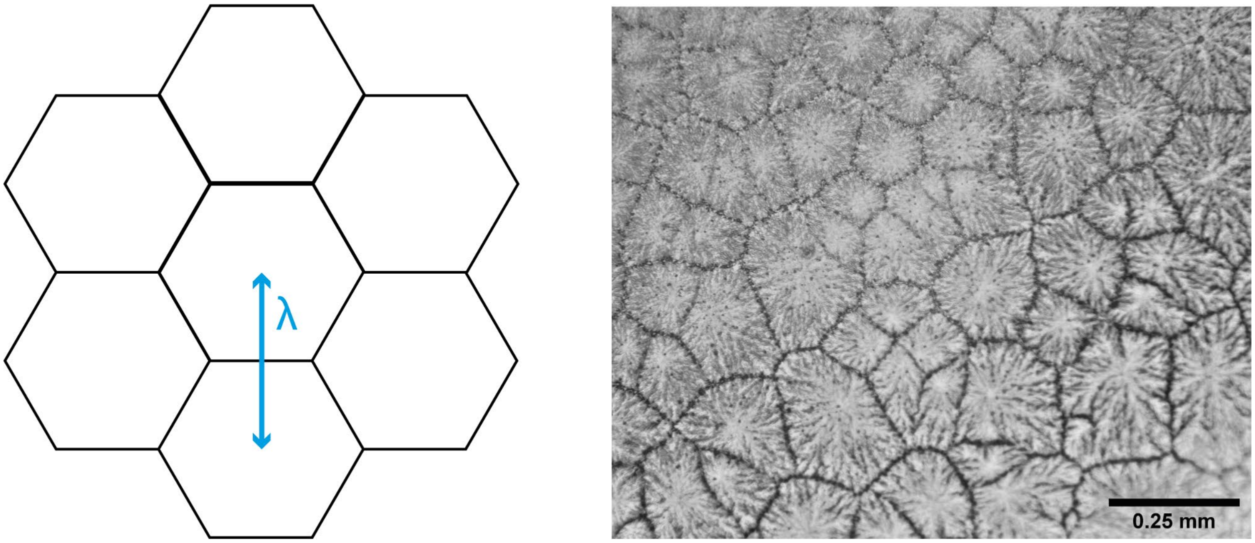

Figure S7: (Left): Cell wavelength (λ) for hexagonal cell-like structures. (Right): Cell-like structures formed in the central crystalline zone of a FBS droplet dried on a glass slide at 30ºC.

Effect of Temperature on Crystalline Growth

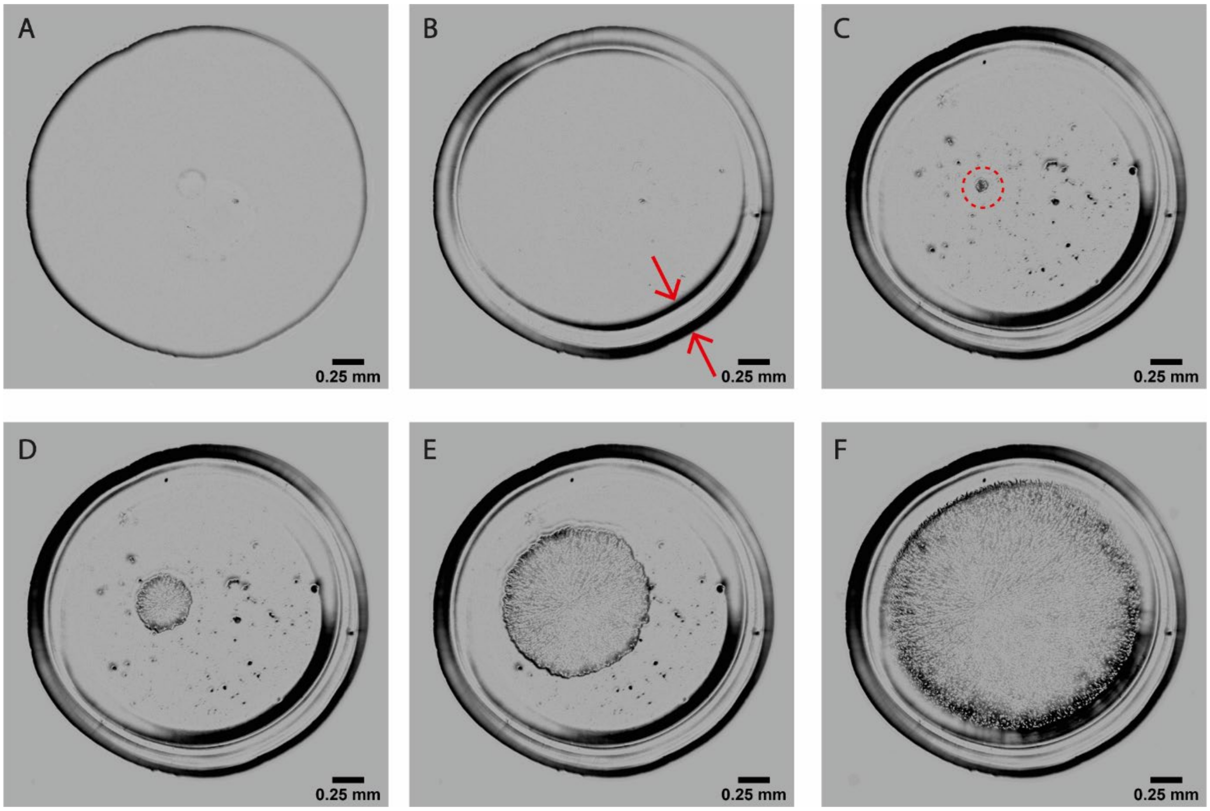

Figure S8: Desiccation stages showing the top-view morphology of a FBS droplet evaporating at 20°C. A)The liquid droplet immediately after deposition on the substrate; B) formation of the glassy protein ring near the periphery of the drop; C) crystal nucleation (564 sec), D-E) crystal growth in a circular pattern (584 and 634 sec respectively), as a single crystal and F) final pattern formation. The background has been subtracted in all the images.

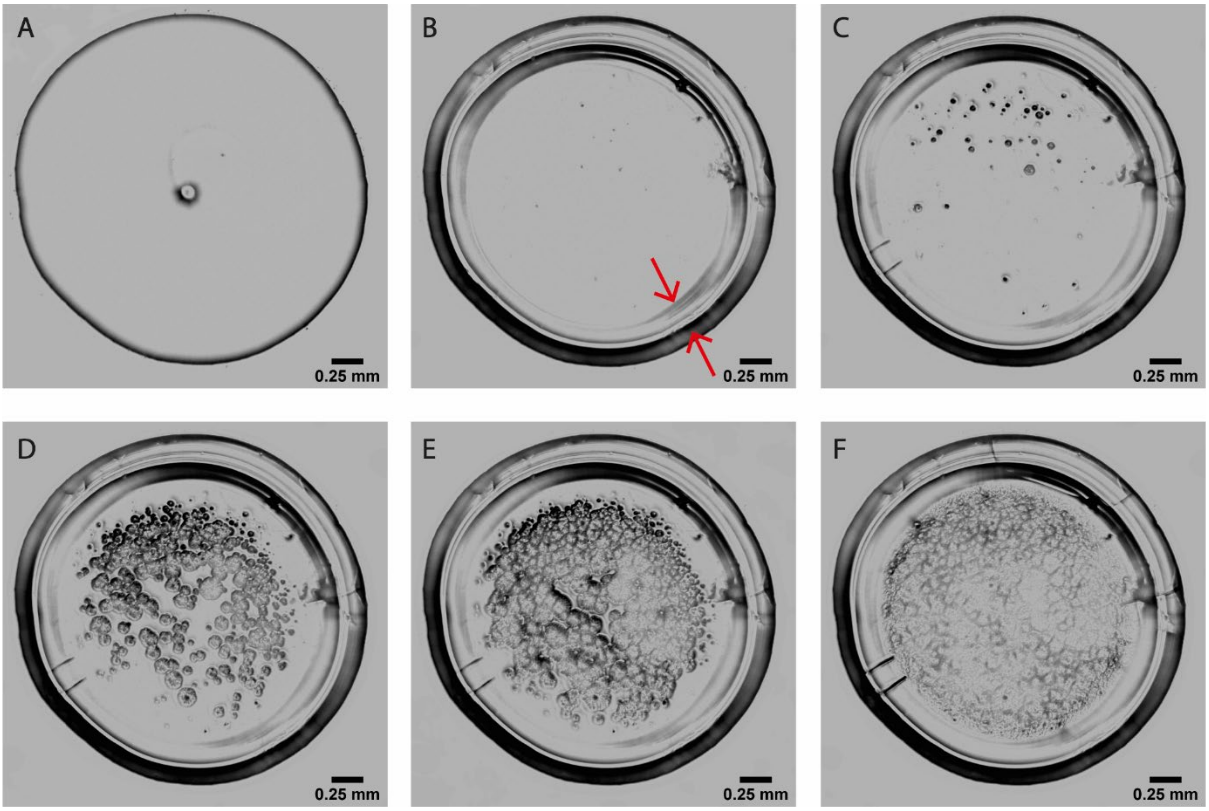

Figure S9: Top-view images showing the desiccation of a FBS droplet evaporating at 25ºC. A) The droplet after deposition on the substrate; B) formation of the protein ring on the periphery of the droplet (510 sec); C) crystal nuclei forming in different regions of the drying drop along with crack propagation on the periphery (562 sec); D-E) crystal growth and propagation of the crystalline area (566 and 570 sec); F) the final desiccated deposit. The background has been subtracted in all the images.

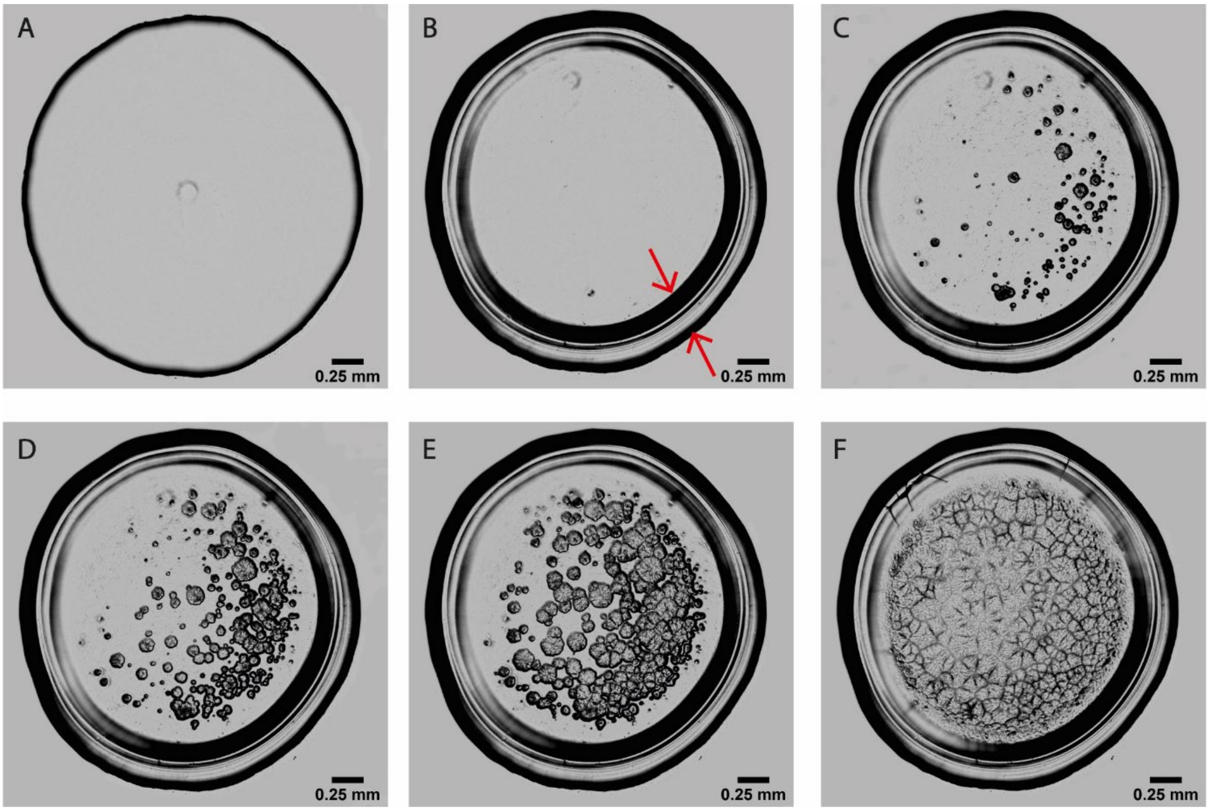

Figure S10: Desiccation of a FBS droplet at 30ºC. A) the deposited droplet on the substrate; B) formation of peripheral protein ring (300 sec); C) crystal nucleation when individual nuclei form in various sites in the desiccating drop (316 sec); D-E) show the crystal growth and the merging of the formed crystals (317 and 318 sec); F) the final desiccation pattern, including cracks on the gel. The background has been subtracted in all the images.

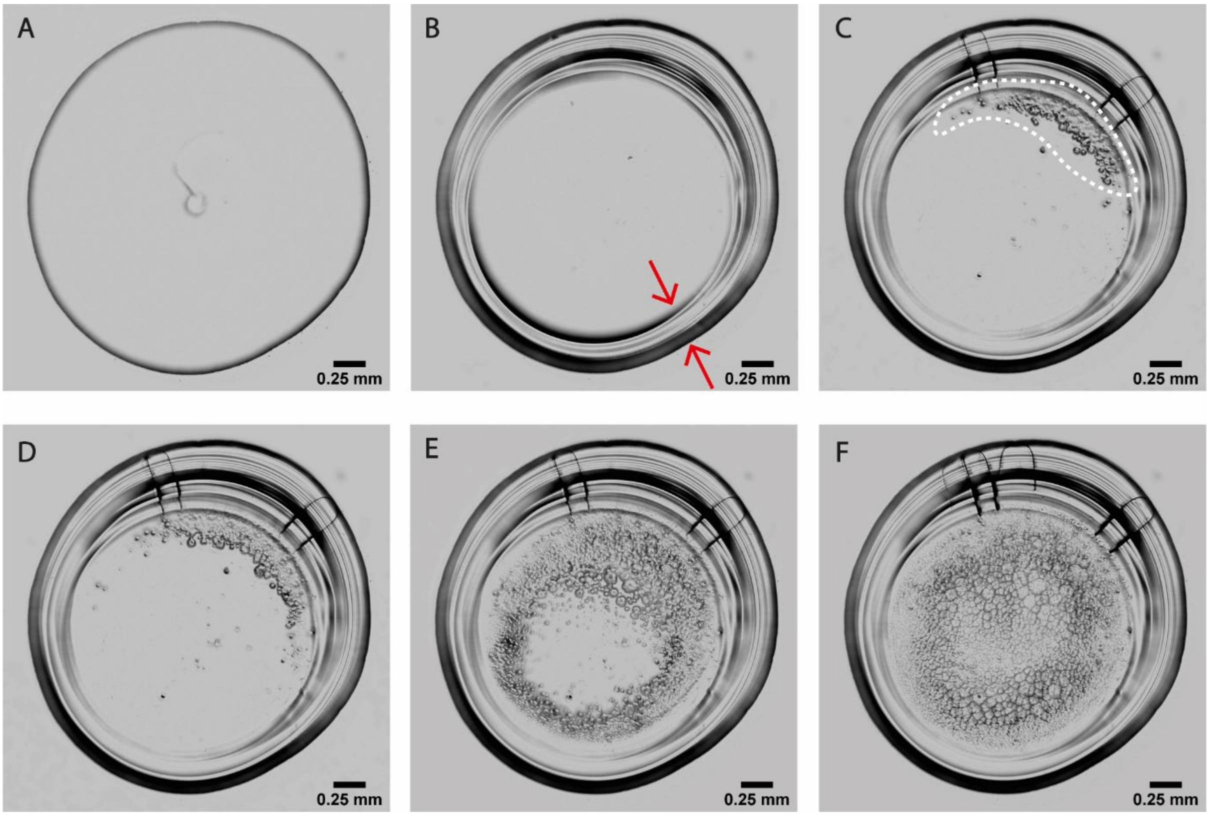

Figure S11: Desiccation of a FBS droplet at 35ºC. A) The liquid droplet on the glass slide after deposition; B) formation of the protein ring on the periphery (163 sec); C) crystal nucleation in an arc-like pattern and crack formation on the periphery (199 sec); D-E) crystal growth (200 and 202 sec respectively); F) the final desiccation pattern. The background has been subtracted in all the images.

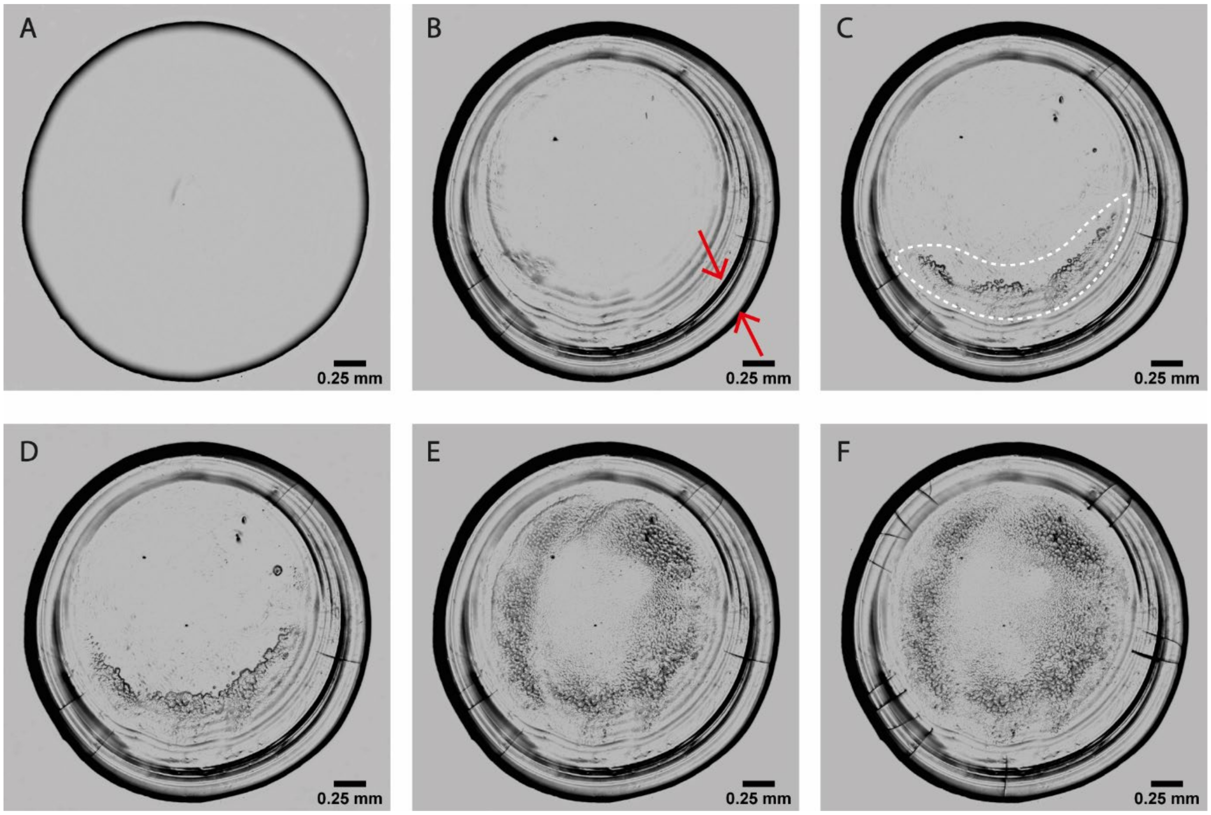

Figure S12: Desiccation of a FBS droplet at 40ºC. A) The liquid droplet immediately post deposition on the substrate; B) completion of the ring formation on the periphery (110sec); C) crystal nucleation in an arc-like pattern (115sec); D-E) crystal growth (116 and 118 sec); F) the final desiccation pattern. The background has been subtracted in all the images.

REFERENCES

1. Sett, A.; Ayushman, M.; Desgupta, S.; Dasgupta, S. Analysis of the Distinct Pattern Formation of Globular Proteins in the Presence of Micro- and Nanoparticles. *J. Phys. Chem. B* **2018**, *122* (38), 8972–8984.

2. Kralchevsky, P. A.; Nagayama, K. Capillary Forces between Colloidal Particles. *Langmuir* **1994**, *10* (1), 23–36.

3. Buschow, K. H. . *Encyclopedia of Materials: Science and Technology*; 2001; Vol. 10.

4. Kaneko, J.; Harvey, J.; Bruss, M. *Clinical Biochemistry of Domestic Animals, 6th Edition*; 2008.

5. Orzheshkovskyi, V. V.; Trishchynska, M. A. Ceruloplasmin: Its Role in the Physiological and Pathological Processes. *Neurophysiology* **2019**, *51* (2), 141–149.

6. Pruzanski, W.; Platts, M. E. Serum and Urinary Proteins, Lysozyme (Muramidase), and Renal Dysfunction in Mono- and Myelomonocytic Leukemia. *J. Clin. Invest.* **1970**, *49* (9), 1694–1708.

7. Israelachvili, J. N. *Intermolecular and Surface Forces, Third Edition*; 2011; Vol. 59.

8. Goehring, L.; Nakahara, A.; Dutta, T.; Kitsunezaki, S.; Tarafdar, S. *Desiccation Cracks and Their Patterns*; Wiley-VCH Verlag GmbH & Co. KGaA: Weinheim, Germany, 2015.

9. Wolf, M.; Gulich, R.; Lunkenheimer, P.; Loidl, A. Broadband Dielectric Spectroscopy on Human Blood. *Biochim. Biophys. Acta - Gen. Subj.* **2011**, *1810* (8), 727–740.

10. Guslisty, A. A.; Malomuzh, N. P.; Fisenko, A. I. Optimal Temperature for Human Life

Activity. *Ukr. J. Phys.* **2018**, *63* (9), 809–815.

11. Armstrong, J. K.; Wenby, R. B.; Meiselman, H. J.; Fisher, T. C. The Hydrodynamic Radii of Macromolecules and Their Effect on Red Blood Cell Aggregation. *Biophys. J.* **2004**, *87* (6), 4259–4270.

12. Roth, C. M.; Neal, B. L.; Lenhoff, A. M. Van Der Waals Interactions Involving Proteins. *Biophys. J.* **1996**, *70* (2 I), 977–987.

13. Rixman, M. A.; Dean, D.; Macias, C. E.; Ortiz, C. Nanoscale Intermolecular Interactions between Human Serum Albumin and Alkanethiol Self-Assembled Monolayers. *Langmuir* **2003**, *19* (15), 6202–6218.

14. Leite, F. L.; Bueno, C. C.; Da Róz, A. L.; Ziemath, E. C.; Oliveira, O. N. *Theoretical Models for Surface Forces and Adhesion and Their Measurement Using Atomic Force Microscopy*; 2012; Vol. 13.

15. Chhasatia, V. H.; Sun, Y. Interaction of Bi-Dispersed Particles with Contact Line in an Evaporating Colloidal Drop. *Soft Matter* **2011**, *7* (21), 10135–10143.

16. Sarangapani, P. S.; Hudson, S. D.; Migler, K. B.; Pathak, J. A. The Limitations of an Exclusively Colloidal View of Protein Solution Hydrodynamics and Rheology. *Biophys. J.* **2013**, *105* (10), 2418–2426.

17. Boström, M.; Tavares, F. W.; Finet, S.; Skouri-Panet, F.; Tardieu, A.; Ninham, B. W. Why Forces between Proteins Follow Different Hofmeister Series for PH above and below PI. *Biophys. Chem.* **2005**, *117* (3), 217–224.

18. Efstratiou, M.; Christy, J.; Sefiane, K. Crystallization-Driven Flows within Evaporating

Aqueous Saline Droplets. *Langmuir* **2020**, *36* (18), 4995–5002.

19. Desarnaud, J.; Derluyn, H.; Carmeliet, J.; Bonn, D.; Shahidzadeh, N. Metastability Limit for the Nucleation of NaCl Crystals in Confinement. *J. Phys. Chem. Lett.* **2014**, *5* (5), 890–895.

20. Chang, Y. C.; Myerson, A. S. The Diffusivity of Potassium Chloride and Sodium Chloride in Concentrated, Saturated, and Supersaturated Aqueous Solutions. *AIChE J.* **1985**, *31* (6), 890–894.

21. Mayén-Mondragón, R.; Yánez-Limón, J. .; Palomares, P.; Sosa, M.; Bernal-Alvarado, J. Thermal Diffusivity of Human Serum and Plasma. *J. Phys. IV* **2005**, *125*, 725–727.
